# Overexpression of Cyclin E1 or Cdc25A leads to replication stress, mitotic aberrancies, and increased sensitivity to replication checkpoint inhibitors

**DOI:** 10.1038/s41389-020-00270-2

**Published:** 2020-10-07

**Authors:** Yannick P. Kok, Sergi Guerrero Llobet, Pepijn M. Schoonen, Marieke Everts, Arkajyoti Bhattacharya, Rudolf S. N. Fehrmann, Nathalie van den Tempel, Marcel A. T. M. van Vugt

**Affiliations:** Department of Medical Oncology, University Medical Center Groningen, University of Groningen, Hanzeplein 1, 9713GZ Groningen, The Netherlands

**Keywords:** Oncogenes, DNA replication, Mitosis

## Abstract

Oncogene-induced replication stress, for instance as a result of Cyclin E1 overexpression, causes genomic instability and has been linked to tumorigenesis. To survive high levels of replication stress, tumors depend on pathways to deal with these DNA lesions, which represent a therapeutically actionable vulnerability. We aimed to uncover the consequences of Cyclin E1 or Cdc25A overexpression on replication kinetics, mitotic progression, and the sensitivity to inhibitors of the WEE1 and ATR replication checkpoint kinases. We modeled oncogene-induced replication stress using inducible expression of Cyclin E1 or Cdc25A in non-transformed RPE-1 cells, either in a *TP53* wild-type or *TP53*-mutant background. DNA fiber analysis showed Cyclin E1 or Cdc25A overexpression to slow replication speed. The resulting replication-derived DNA lesions were transmitted into mitosis causing chromosome segregation defects. Single cell sequencing revealed that replication stress and mitotic defects upon Cyclin E1 or Cdc25A overexpression resulted in genomic instability. ATR or WEE1 inhibition exacerbated the mitotic aberrancies induced by Cyclin E1 or Cdc25A overexpression, and caused cytotoxicity. Both these phenotypes were exacerbated upon p53 inactivation. Conversely, downregulation of Cyclin E1 rescued both replication kinetics, as well as sensitivity to ATR and WEE1 inhibitors. Taken together, Cyclin E1 or Cdc25A-induced replication stress leads to mitotic segregation defects and genomic instability. These mitotic defects are exacerbated by inhibition of ATR or WEE1 and therefore point to mitotic catastrophe as an underlying mechanism. Importantly, our data suggest that Cyclin E1 overexpression can be used to select patients for treatment with replication checkpoint inhibitors.

## Introduction

A common hallmark of cancer is the acquisition of genomic gains and losses as well as complex genomic re-arrangements, collectively termed genomic instability^1^. Genomic instability drives intra-tumor heterogeneity, which is an important factor underlying therapy failure^2^. Stalling or slowing of replication, commonly referred to as ‘replication stress’, is increasingly considered to be an important factor in fueling genomic instability in cancer^3,4^. Although there are various factors that induce replication stress, a common cause in the context of cancer is the increased activity or elevated expression of oncogenes^4–6^.

Amplification of *CCNE1* (encoding for Cyclin E1) is frequently observed in genomically instable tumors, including high-grade serous ovarian cancer and triple negative breast cancer (TNBC)^7–12^, and has been associated with a poor prognosis in these and various other tumor types^13–16^. *CCNE1* amplification has been linked to induction of replication stress, by causing collisions between the replication and transcription machineries^17^, and by triggering aberrant firing of replication origins, which subsequently leads to depletion of the nucleotide pool^3,17^. Combined, these effects can lead to stalling or collapse of replication forks^4^. Oncogene-induced replication stress triggers a DNA damage response, with ensuing genetic pressure to inactivate *TP53*^6^. In good agreement with these observations, Cyclin E1 overexpression was demonstrated to exclusively induce genome instability in tumors lacking functional p53^18–20^.

Multiple oncogenic events were shown to exert their effects on DNA replication through direct or indirect elevation of Cyclin-dependent kinase-2 (CDK2) activity^21–24^. CDK2 activity is important in regulating the ‘firing’ of replication origins^17,25,26^, and is primarily controlled by the abundance of its Cyclin partner. Indeed, overexpression of Cyclin E1 elevates CDK2 activity^26^. Importantly, CDK2 activity—determined by inhibitory phosphorylation of Tyr15^27^—is catalyzed by the WEE1 kinase^28,29^, and can be removed by the Cdc25A phosphatase^30^. In line with this notion, overexpression of Cdc25A has been shown to result in CDK2 hyperactivation^27^. Consequently, overexpression of either CCNE1 or Cdc25A leads to aberrant firing of replication origins and triggers a replication stress response^17^.

Since replication stress hampers cell growth, cancers harboring oncogene-induced replication stress have apparently adapted to cope with replication stress. In order to find better treatments for tumors with oncogene-induced replication stress, it could be of great clinical interest to target pathways that allow tumors to deal with replication stress. Particularly interesting in this context are cell cycle checkpoint kinases. Previously, tumor cells with genome instability due to defective homologous recombination were shown to depend on the ATR and WEE1 replication checkpoint kinases for their survival^31,32^. Furthermore, lymphomas driven by *MYC* amplification—which triggers profound replication stress—were shown to be highly sensitive to CHK1 inhibition^33^. In order to optimally implement cell cycle checkpoint inhibitors in cancer treatment, and identify patients who benefit from such treatments, it is essential to understand how cancer cells deal with replication stress, and uncover the mechanisms underlying checkpoint kinase inhibitor-mediated cytotoxicity in cancer cells.

It is increasingly apparent that the resolution of replication stress is highly complex and not restricted to S-phase. Indeed, resolving late-stage replication intermediates was observed even when cells had already entered mitosis^34,35^. In line with these observations, our recent data underscored the notion that PARP inhibitor-induced replication-mediated DNA lesions are transmitted into mitosis, and cause chromosome segregation defects and mitotic failure^32^. Whether these findings hold true for other sources of replication stress is currently unknown. In this study, we assessed whether oncogene-induced replication stress as a result of Cyclin E1 or Cdc25A overexpression affects mitotic behavior of tumor cells and genome instability. Additionally, we studied whether replication stress can be targeted through inhibition of the cell cycle checkpoint kinases WEE1 and ATR.

## Results

### Overexpression of cyclin E1 or Cdc25A leads to slower replication kinetics and mitotic defects

**Fig. 1:**
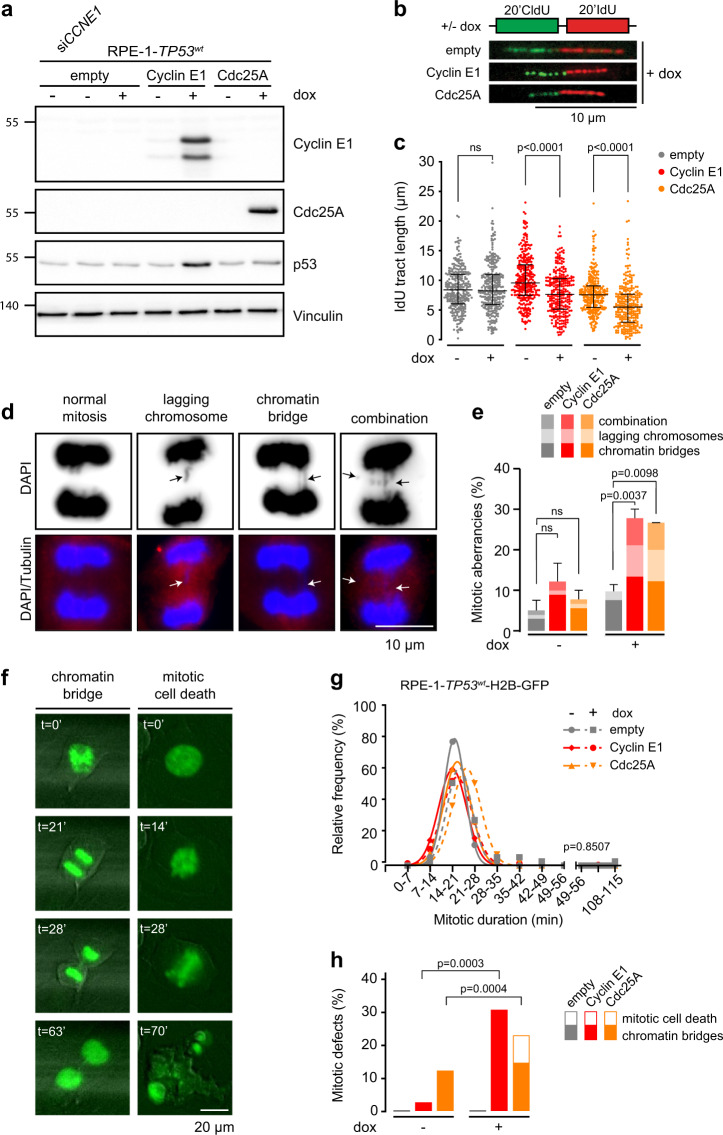
Cdc25A or Cyclin E1 overexpression leads to replication stress. **a** RPE-1-*TP53*^wt^ cells were engineered to overexpress empty, Cyclin E1 or Cdc25A constructs in a doxycycline-inducible manner. Immunoblot shows Cyclin E1, Cdc25A, p53, and Vinculin protein levels at 48 h after addition of doxycycline (dox). **b** Cells were treated with doxycycline for 48 h, were subsequently labeled for 20 min with CldU (25 µM) and for 20 min with IdU (250 µM). Representative DNA fibers from doxycycline-treated cells are shown. Scale bar measures 10 µm. **c** Quantification of IdU DNA fiber lengths as described in panel **b**. At least 266 fibers were analyzed. Graphs show individual data points, median and interquartile range. *p*-values were calculated using the Mann–Whitney *U* test**. d** Examples of chromatin bridges and lagging chromosomes. Cells were stained with α-Tubulin (red) and counterstained with DAPI (blue). Scale bar indicates 10 µm. **e** Quantification of anaphase and telophase cells containing chromatin bridges and/or lagging chromosomes. The bars represent the mean and standard error or the mean (SEM) from three experiments, *n* > 25 per experimental condition; *p*-values were calculated using two-tailed Student’s *t*-test. **f** Representative examples of mitotic aberrancies observed in RPE-1*-TP53*^wt^ cells transduced with H2B-EGFP using live-cell microscopy. Scale bar represents 20 µm. **g** Duration of mitosis as measured by nuclear envelope breakdown to anaphase. Cells were pre-treated with doxycycline for 24 h and subsequently followed with live-cell microscopy using 7 min intervals for the duration of 48 h. *p*-value was calculated using a Kruskal–Wallis test. **h** Quantification of aberrant mitoses in cells from panel **h**. *p*-values were calculated using absolute values, using Mann–Whitney *U* test.

Cyclin E1 is often found to be overexpressed in cancers, specifically in TNBCs and high-grade ovarian cancers^7,8^, which is accompanied by higher CCNE1 mRNA expression levels in these cancers (Supplementary Fig. 1A). To study the effects of Cyclin E1 overexpression on replication kinetics, we engineered hTERT-immortalized human retinal pigmented epithelial (RPE-1) cells to overexpress a truncated oncogenic version of Cyclin E1 in a doxycycline-dependent manner. Doxycycline treatment resulted in a ~70-fold increased expression of Cyclin E1 compared to endogenous levels (Fig. 1a and Supplementary Fig. 1B). In parallel, we evaluated the effects of Cdc25A overexpression, as this protein also leads to CDK2 hyperactivation, albeit through an alternative mechanism (Fig. 1a). To test whether overexpression of Cyclin E1 or Cdc25A affected replication dynamics, cells were treated with doxycycline for 48 h, and cells were subsequently incubated with thymidine analogs CldU and IdU to label ongoing replication (Fig. 1b). Single DNA fibers were analyzed to measure replication kinetics. The IdU fiber tract length was reduced by 28% in Cyclin E1-overexpressing cells and 31% in Cdc25A-overexpressing cells, indicating a robust reduction of ongoing DNA synthesis speed compared to parental RPE-1-*TP53*^*wt*^ cells (Fig. 1c).

We next tested whether the observed replication stress resulted in mitotic aberrancies. To this end, we quantified the amount of chromatin bridges and lagging chromosomes during anaphase and telophase at 48 h after induction of Cyclin E1 or Cdc25A overexpression in RPE-1-*TP53*^wt^ cells (Fig. 1d). Doxycycline-induced Cyclin E1 or Cdc25A overexpression resulted in a 3-fold increase in mitotic aberrancies when compared to control cells (Fig. 1e). Both chromatin bridges and lagging chromosomes were increased in Cyclin E1 and Cdc25A-overexpressing (Fig. 1e). A third type of mitotic aberration, ultra-fine bridges^36^, was only increased in Cdc25A-overexpressing cells (26% vs. 14%) but not in Cyclin E1-overexpressing cells (11% vs. 14%) (Supplementary Fig. 1C). To further investigate the mitotic aberrancies induced by oncogene-induced replication stress, RPE-1-*TP53*^wt^ cells overexpressing Cyclin E1 or Cdc25A were analyzed by live-cell microscopy. To this end, cells were transduced with EGFP-tagged Histone-H2B, treated with doxycycline to induce overexpression of Cyclin E1 or Cdc25A and were then followed for the duration of 48 h, capturing images every 7 min using live cell microscopy (Fig. 1f). Overexpression of Cyclin E1 or Cdc25A did not significantly affect mitotic duration as measured by the time between nuclear envelope break-down (NEB) and anaphase entry (Fig. 1g), but did increase the frequency of mitotic aberrancies (23% in Cyclin E1-overexpressing cells and 19% in Cdc25A-overexpressing cells vs. 12% and 3% in respective control-treated cells, Fig. 1h). Combined, these data show that both Cyclin E1 and Cdc25A-induced replication stress results in the formation of chromatin bridges and lagging chromosomes, whereas Cdc25A overexpression also increases ultra-fine bridge formation.

### TP53 mutation exacerbates replication stress and mitotic defects

**Fig. 2:**
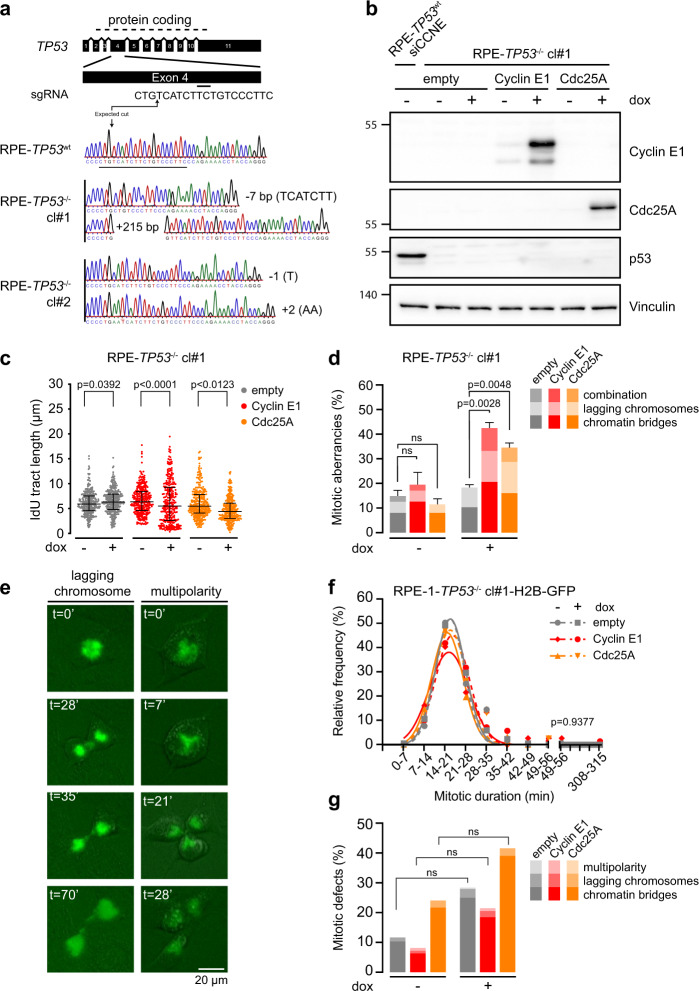
Mutation of TP53 exacerbates replication stress and mitotic defects. **a** Schematic overview of CRISPR/Cas9 gene targeting in *TP53* gene. The exon map and protein coding are based on Emsembl entry ENSG00000141510. Placement of the sgRNA sequence is indicated with a horizontal line under exon 4 and the wild type sequence. Sanger sequencing shows that the gRNA targeting exon 4 induced a −7 bp deletion and a +215 bp insertion in RPE-*TP53*^−/−^ cl#1 and a −1 deletion and +2 insertion in RPE-*TP53*^−/−^ cl#2, leading to frame-shifts in *TP53*. **b** RPE-1-*TP53*^−/−^ cl#1 cells were engineered to overexpress empty, Cyclin E1 or Cdc25A constructs in a doxycycline-inducible manner. Immunoblot shows Cyclin E1, Cdc25A, p53, and Vinculin protein levels at 48 h after addition of doxycycline (dox). RPE-1-*TP53*^wt^ cells were used as a positive control for p53. **c** Cells were treated with doxycycline for 48 h, and were then labeled for 20 min with CldU (25 µM) and subsequently for 20 min with IdU (250 µM). Per condition at least 279 fibers were analyzed. Graphs show individual data points, median and interquartile range. *p*-values were calculated using the Mann–Whitney *U* test. **d** Quantification of anaphase or telophase cells containing chromatin bridges or lagging chromosomes. The bars represent mean and standard error or the mean (SEM) from three experiments, *n* > 25 per experimental condition; *p*-values were calculated using two-tailed Student’s *t*-test. **e** Representative examples of mitotic aberrancies observed in RPE-1*-TP53*^−/−^ cells transduced with H2B-EGFP cells using live-cell microscopy. Scale bar represents 20 µm. **f** Duration of mitosis as measured by NEB breakdown to anaphase. Cells were pre-treated for 24 h with doxycycline and subsequently followed with live-cell microscopy using 7 min intervals for the duration of 48 h. *p*-value was calculated using a Kruskal–Wallis test. **g** Quantification of aberrant mitoses in cells from panel **f**. *p*-values were calculated using absolute values, using Mann–Whitney *U* test.

Since oncogene expression in genomically instable cancers is frequently associated with loss of *TP53*, we used CRISPR/Cas9 to mutate *TP53* in RPE-1 cells (Fig. 2a). We selected two *TP53*-mutant clones and introduced the doxycycline-inducible Cyclin E1 and Cdc25A constructs or an empty vector to assess how p53-negative cells behave upon overexpression of these oncogenes (Fig. 2b, Supplementary Fig. 2A, B). Compared to endogenous Cyclin E1 levels, doxycycline treatment increased the expression by ~60-fold in clone #1 and ~38-fold in clone #2 (Supplementary Fig. 2C). Like in *TP53*-wt cells, overexpression of Cyclin E1 or Cdc25A in RPE-1-*TP53*^−/−^ cells reduced IdU tract length by 7–53% compared to untreated conditions (Fig. 2c, Supplementary Fig. 2D).

We next analyzed the amounts of mitotic aberrancies. In line with previous reports, RPE-1-*TP53*^−/−^ cells showed higher basal frequencies of mitotic aberrancies when compared to RPE-1-*TP53*^wt^ cells (17% vs. 4%, Figs. 1d and 2d, Supplementary Fig. 2E)^37^. The percentage of mitotic aberrancies increased from 17% to 41.1% in Cyclin E1-overexpressing cells and to 33.3% in Cdc25A-overexpressing cells (Fig. 2d). We did not observe an increase in the amount of ultra-fine bridges upon Cyclin E1 or Cdc25A overexpression in *TP53-*mutated cells (Supplementary Fig. 2F).

To confirm that the absence of p53 expression leads to elevated amounts of mitotic defects, we analyzed H2B-EGFP-expressing cells using live-cell imaging (Fig. 2e). Analogous to previous observations in *TP53*^*wt*^ cells, overexpression of Cyclin E1 or Cdc25A in RPE-1-*TP53*^−/−^-H2B-EGFP cells did not result in a significant change in the duration of mitosis (Fig. 2f). We did observe more mitotic defects at baseline in RPE-1-*TP53*^−/−^ cells than in RPE-1-*TP53*^*wt*^ cells (Figs. 1h and 2e). Although not statistically significant, Cyclin E1 and Cdc25A overexpression in *TP53*^−/−^ cells did increase the percentage of mitotic defects (Fig. 2g). These data underscore that replication stress and mitotic errors are increased upon *TP53* inactivation, and point towards exacerbation of this phenotype upon Cyclin E1 and Cdc25A overexpression.

### Cyclin E1 or Cdc25A overexpression induces genomic instability

**Fig. 3:**
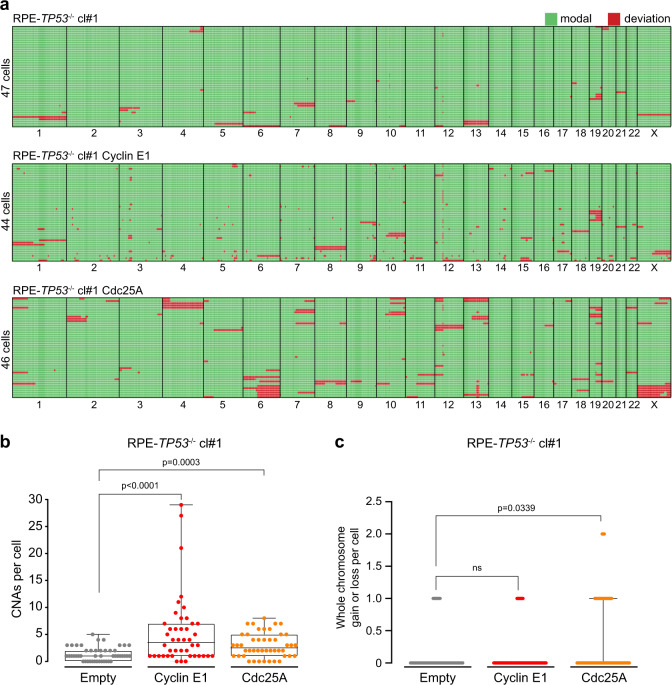
Cyclin E1 or Cdc25A overexpression induces genomic instability. **a** Genome-wide copy number deviation plots of RPE-*TP53*^−/−^ cl#1 empty (*n* = 47), RPE-*TP53*^−/−^ cl#1 -Cyclin E1 (*n* = 44) and RPE-*TP53*^−/−^ cl#1 **-**Cdc25A cells (*n* = 46). Cells were treated with doxycycline for 120 h. After single cell sorting, genomic DNA was harvested for single-cell whole genome sequencing (sc-WGS). Each panel displays the individual cells in rows, and the chromosomes numbers from 1-X in columns. The modal copy number state is pictured in green, deviations of the modal copy number state, both focal and whole-chromosome, are colored red). **b** Copy-number alterations (CNAs) per cell were calculated according to the modal state. Medians with interquartile range are depicted and statistical analyses were performed using a One-sided Mann–Whitney *U* test. **c** whole numerical chromosomes per cell were counter per single cell. Medians with interquartile range are depicted and statistical analyses were performed using a one-sided Mann–Whitney *U* test.

Elevated levels of Cyclin E1 have previously been associated to structural chromosome defects^20,38^. Moreover, overexpression of both Cyclin E1 and Cdc25A has been shown to result in loss of specific genomic regions^39–41^. Furthermore, a mouse model of Cyclin E1 overexpression resulted in tumors with genomic instability^42^. Indeed, we also observed correlations between mRNA expression of *CCNE1* or *CDC25A* and copy number load in various tumor types (Supplementary Fig. 3A–C). However, since some of these observations could be explained by indirect effects, we employed single-cell whole genome sequencing to assess if and how the observed chromosome segregation defects upon short-term overexpression of Cyclin E1 or Cdc25A in RPE-1-*TP53*^−/−^ cells translate into structural or numerical chromosome aberrations^43^. Of note, we observed genomic deviations that arose in the process of engineering the *TP53*^*−/−*^ cell lines, underscoring the importance of analyzing multiple clones (Supplementary Fig. 4A). Importantly, we observed increased numbers of focal copy number alterations (CNAs) upon induction of Cyclin E1 or Cdc25A overexpression for 5 days (Fig. 3a, b and Supplementary Fig. 4B–D). This increase was statistically significant in RPE-1-*TP53*^−/−^ clone #1, but not in clone #2, possibly due to the limited number of cells that were analyzed, a relatively short treatment time, and lower levels of overexpression in clone #2 (Supplementary Fig. 2C). In RPE-1-*TP53*^−/−^ clone #1, Cyclin E1 overexpression resulted in more copy number aberrations compared to empty vector control (Fig. 3b and Supplementary Fig. 4C, whereas Cdc25A overexpression resulted in more whole chromosome aberrations (Fig. 3c and Supplementary Fig. 4D). These data suggest that the increased mitotic errors upon Cyclin E1 or Cdc25A overexpression translate into genomic instability.

### Cyclin E1 and Cdc25A-induced mitotic aberrancies are exacerbated upon treatment with ATR and WEE1 inhibitors

**Fig. 4:**
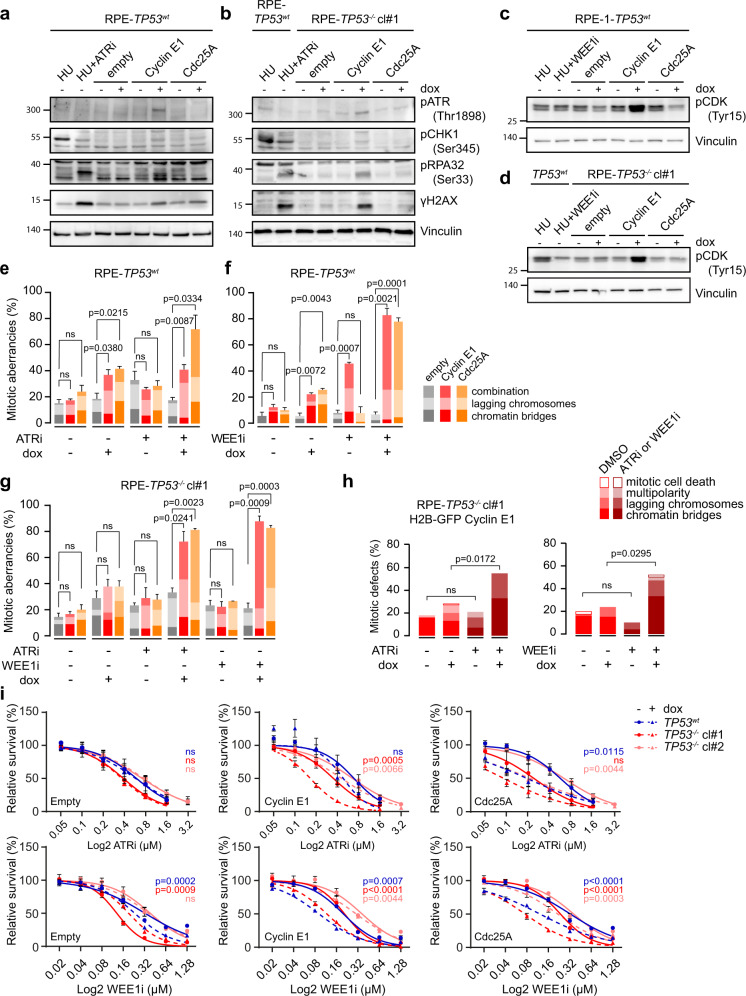
ATR and WEE1 inhibition cause mitotic aberrancies. **a**, **b** RPE-*TP53*^wt^ (panel **a**) and RPE-*TP53*^−/−^ cl#1 (panel **b**) cells were treated with doxycycline for 72 h to induce overexpression of Cyclin E or Cdc25A. Control cells (RPE-*TP53*^wt^) were then left untreated or were treated with ATR inhibitor (ATRi, VE-822, 1 µM) for 2 h, followed by a 6 h treatment with hydroxyurea (HU, 1 mM) and immunoblotted for ATR-response proteins pATR, pCHK1, pRPA, and γH2AX, and for WEE1-response marker pCDK (Tyr15). Vinculin serves as a loading control. **c**, **d** RPE-*TP53*^wt^ (panel **c**) and RPE-*TP53*^−/−^ cl#1 (panel **d**) were treated with doxycycline for 72 h to induce overexpression of Cyclin E or Cdc25A. Control cells (RPE-*TP53*^wt^) were then left untreated or were treated with ATR inhibitor (ATRi, VE-822, 1 µM) for 2 h, followed by a 6 h treatment with hydroxyurea (HU, 1 mM) and immunoblotted for WEE1 response protein pCDK (Tyr15). **e** RPE-1-*TP53*^wt^ cells induced to express Cyclin E1 or Cdc25A were treated with ATR inhibitor (ATRi, VE-822, 0.25 µM) for 8 h as indicated. The percentages of anaphase or telophase cells containing chromatin bridges or lagging chromosomes were quantified. The bars represent mean and standard error or the mean (SEM) from three experiments, *n* > 25 per condition; *p*-values were calculated using two-tailed Student’s *t*-test. **f** RPE-1-*TP53*^wt^ cells induced to express Cyclin E1 or Cdc25A were treated with WEE1 inhibitor (WEE1i, MK-1775, 0.1 µM) for 8 h if indicated. The percentages of anaphase or telophase cells containing chromatin bridges or lagging were quantified. The bars represent mean and SEM from three experiments, *n* > 25 per experimental condition; *p*-values were calculated using two-tailed Student’s *t*-test. **g** RPE-1-*TP53*^−/−^ cl#1 cells induced to express Cyclin E1 or Cdc25A were treated as in panels **e** and **f**. The percentages of anaphase or telophase cells containing chromatin bridges or lagging chromosomes were quantified. The bars represent mean and SEM from three experiments, *n* > 25 per experimental condition; The *p*-values were calculated by one-way ANOVA (*p* < 0.0001) and followed by Sidak’s multiple comparison test. **h** Percentage of RPE-1-*TP53*^−/−^ cl#1 -Cyclin E1-H2B-EGFP cells that showed aberrant mitoses. Cells were pre-treated for 24 h with doxycycline. Cells were then treated with ATR inhibitor (VE-822, 0.25 µM) or WEE1 inhibitor (MK-1775, 0.1 µM), and subsequently followed with live-cell microscopy using 7 min intervals for 48 h. *p*-values were calculated using a Mann–Whitney *U* test. **i** RPE-1-*TP53*^wt^ and RPE-1-*TP53*^−/−^ cl#1 cell lines were induced to express Cyclin E1 or Cdc25A, and were treated for 3 days with ATR inhibitor (VE-822) in a range from 0 to 3.2 µM, or WEE1 inhibitor (MK-1775) in a range from 0 to 1.28 µM. Subsequently, relative cell survival was assessed using MTT conversion as a proxy. Plots include mean and standard error of the means (SEM) of three biological replicates. Reported *p*-values were calculated by a Student’s *t*-test comparing the area under the curve of doxycycline-untreated samples to the curve of the doxycycline-treated samples.

The observation that Cyclin E1 or Cdc25A overexpression leads to replication stress, mitotic aberrations, and ensuing focal copy number alterations likely indicated that replication-born DNA lesions remain unresolved when cells enter mitosis. Indeed, regardless of *TP53*-status, we observed that Cyclin E1-overexpressing cells, and to a lesser extent Cdc25A-overexpressing cells have increased amounts of the DNA damage marker pH2AX Ser139 (γH2AX) (Fig. 4a, b, Supplementary Fig. 5A, B). These cells also activated a replication stress response mediated by ATR, as measured by pATR Thr1989, pCHK1 Ser345, and pRPA32 Ser33 (Fig. 4a, b) and elevated WEE1 activity as measured by levels of pCDK Tyr15 (Fig. 4c, d, Supplementary Fig. 5A, B). Although the observed activation of the ATR and WEE1 kinases did not completely prevent mitotic errors from occurring, inhibiting this response could enforce premature mitotic entry^44^, thereby exacerbate chromosome segregation errors in Cyclin E1-overexpressing or Cdc25A-overexpressing cells. To test this, we induced overexpression of Cyclin E1 or Cdc25A in RPE-1-*TP53*^wt^ or RPE-1-*TP53*^−/−^ cells for 48 h, and subsequently treated the cells with ATR or WEE1 inhibitors for 8 h. Upon overexpression of Cyclin E1 in RPE-1-*TP53*^wt^ cells, WEE1 inhibition, but not ATR inhibition, resulted in a significant increase of mitotic aberrancies (Fig. 4f, g). In Cd25A-overexpressing RPE-1-*TP53*^wt^ cells, inhibition of ATR and WEE1 both enhanced the frequency of mitotic aberrancies (41.1–72.2% upon ATR inhibition and 25.6–77.8% upon WEE1 inhibition, Fig. 4e, f).

In the RPE-1 *TP53*^−/−^ clones, both ATR and WEE1 inhibition increased the frequency of mitotic aberrancies in Cyclin E1-overexpressing cells (from 37.7% to 72.2% upon ATR inhibition and up to 87.8% upon WEE1 inhibition, Fig. 4g, Supplementary Fig. 5C), and in Cdc25A-overexpressing cells (from 37.7% to 81.1% upon ATR inhibition and to 82.7% upon WEE1 inhibition, Fig. 4g) We did not observe an increase in ultra-fine bridges upon inhibition of ATR or WEE1 in any of the tested conditions (Supplementary Fig. 5D, E).

We next used live cell microscopy to investigate whether chromosome segregation defects induced by ATR or WEE1-inhibition in Cyclin E1-overexpressing RPE-1-*TP53*^−/−^ cells translated into altered mitotic fidelity and duration. Indeed, ATR inhibitor treatment in Cyclin E1-overexpressing RPE-1-*TP53*^−/−^ cells increased the percentage of mitoses with chromatin bridges from 13% to 33%, and increased the percentage of lagging chromosomes from 7% to 22% (Fig. 4g). Similarly, WEE1 inhibition exacerbated the formation of chromatin bridges in Cyclin E1-overexpressing cells from 15% to 33% (Fig. 4g), and increased the percentage of lagging chromosomes from 9% to 14% (Fig. 4g). The induction of mitotic aberration by ATR and WEE1 inhibition was confirmed in RPE-1-*TP53*^−/−^ H2B-EGFP using live cell microscopy (Fig. 4h). ATR nor WEE1 inhibition affected mitotic duration in Cyclin E-overexpressing cells (Supplementary Fig. 5F). To measure premature mitotic upon ATR or WEE1 inhibition, cells were synchronized using a double thymidine block. In line with previous reports, ATR inhibition accelerated entry into mitosis, leading to a burst in mitotic cells^44^, whereas WEE1 inhibition did not^45^ (Supplementary Fig. 5G). These data indicate that ATR inhibition may affect mitotic fidelity by premature mitotic entry, whereas the effects of WEE1 inhibition appear more complex.

### Overexpression of cyclin E1 or Cdc25A results in increased sensitivity to ATR and WEE1 inhibition

Using MTT assays, we next examined whether the enhanced occurrence of mitotic aberrancies upon ATR or WEE1 inhibition in Cyclin E1 or Cdc25A-overexpressing cells is accompanied with increased sensitivity towards ATR or WEE1 inhibition. In line with the absence of increased mitotic aberrancies upon ATR inhibitor treatment in *TP53*^wt^ Cyclin E1-overexpressing cells, we observed that Cyclin E1 overexpression only sensitized RPE-*TP53*^−*/*−^ cells to ATR inhibition (Fig. 4i), indicating that loss of p53 function is required for ATR inhibitor sensitivity in Cyclin E1-overexpressing cells. In contrast, loss of p53 function was not required for WEE1 inhibitor sensitivity in Cyclin E1-overexpressing cells, although it did enhance sensitivity (Fig. 4i). Cdc25A overexpression sensitized both RPE-1-*TP53*^wt^ and RPE-1-*TP53*^−/−^ cells to ATR inhibition as well as to WEE1 inhibition (Fig. 4i). These data indicate that Cyclin E1 or Cdc25A overexpression sensitizes cells to inhibition of the ATR or WEE1 checkpoint kinases.

### Reduction of cyclin E1 levels diminishes replication stress and mitotic errors

**Fig. 5:**
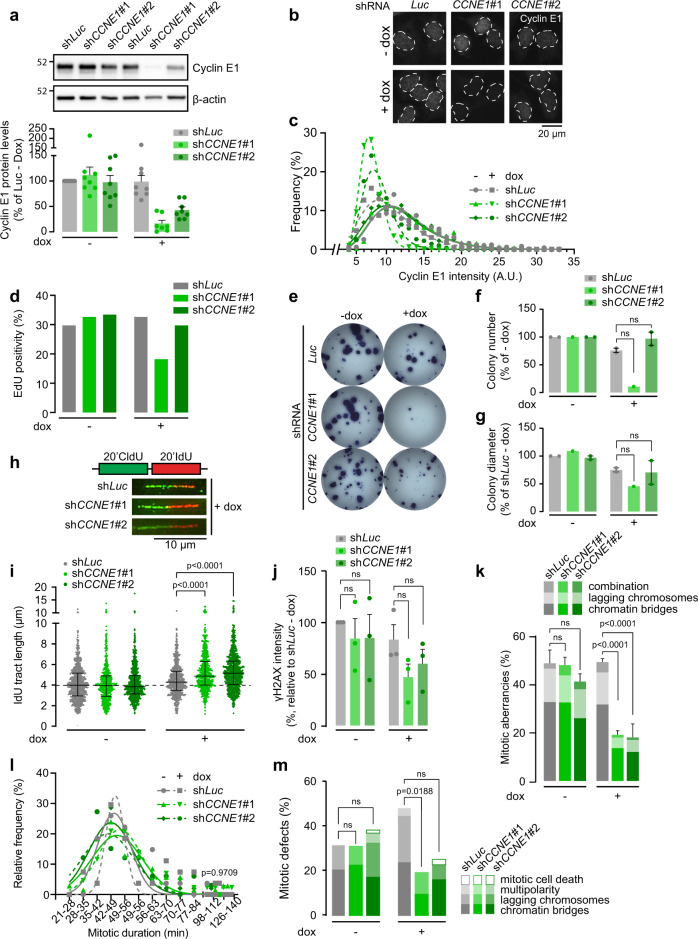
Reducing Cyclin E1 overexpression diminishes replication stress and mitotic errors. **a** HCC1806 cells transduced with inducible Cyclin E1 construct (sh*CCNE1*#1 or sh*CCNE1*#2) or control shRNA (sh*Luc*) were treated with doxycycline for 2 days, and immunoblotted for Cyclin E1 and β-Actin. Cyclin E1 protein levels were measured and normalized to ‘sh*Luc* -DOX’ controls for each experiment. Bar graphs reflect the average and standard deviation from eight independent experiments. **b** Cyclin E1 knock-down after 2 days of doxycycline treatment assessed by immunofluorescence microscopy. The white lines indicate boundaries of nuclei based on DAPI counterstaining. **c** Average staining intensity of Cyclin E1 as shown in panel **b** was categorized and plotted in a histogram. The curve fitted is a log-normal Gaussian distribution. At least 450 nuclei were measured. **d** Percentage of EdU-positive cells after 48 h of doxycycline treatment, measured by flow cytometry. **e** Representative pictures of clonogenic survival of HCC1806 cells. Cells were plated in six-well plates and allowed to attach for 24 h, after which doxycycline was added. After 14 days, surviving colonies were stained. **f**, **g** Colony survival percentages compared to Luc-dox controls **f** and relative average diameter of colonies counted **g** in panel **f**, relative to Luc-dox control. Bars represent the mean and standard error of the mean (SEM) mitotic fraction of two independent experiments. *p*-values were calculated using two-tailed Student’s *t*-test **h** cells were treated with doxycycline for 48 h and sequentially labeled for 20 min with CldU (25 µM) and 20 min with IdU (250 µM). Representative DNA fibers of doxycycline-treated samples are shown. **i** Quantification of IdU DNA fiber lengths as described in panel **h**. Per condition, at least 466 fibers were analyzed and corresponding medians with interquartile range are shown. *p*-value was calculated using Mann–Whitney *U* test. **j** γH2AX intensity as measured by flow cytometry in cells treated with and without doxycycline for 48 h. Means and SEM normalized to the untreated luciferase condition are shown from three biological replicates. **k** Cyclin E1 knock-down was induced by doxycycline treatment for 48 h. Cells were then fixed and the percentage of mitotic aberrancies was quantified. Data represents mean and SEM of three independent experiments; at least 30 mitoses were analyzed for each experimental condition. The *p*-values were calculated by one-way ANOVA (*p* < 0.0001) and followed by Sidak’s multiple comparison test. **l** Duration of mitosis as measured by NEB breakdown to anaphase. HCC1806 H2B-EGFP cells were pre-treated for 48 h with doxycycline and subsequently followed with live-cell microscopy in 7 min intervals for the duration of 48 h. *p*-value was calculated using a Kruskal–Wallis test. and subsequently followed with live-cell microscopy using 7 min intervals for 48 h. Duration of mitosis is shown. **m** Quantification of aberrant mitoses in cells from panel **l**. *p*-values were calculated using absolute values, using Mann–Whitney *U* test.

To test whether high expression levels of Cyclin E1 influenced DNA replication kinetics and sensitivity of cancer cells to ATR and WEE1 inhibitor, we aimed to downregulate Cyclin E1 expression in TNBC cancer cells. We first tested the sensitivity to ATR and WEE1 inhibition in three TNBC cell lines (MDA-MB-157, HCC1806, and HCC1569) that have a 19q12 amplification which encompasses the *CCNE1* gene^46^. HCC1806, and to a lesser extent HCC1569, were sensitive to both ATR and WEE1 inhibition (Supplementary Fig. 6A, B). MDA-MB-157 cells did not display notable sensitivity to either drug (Supplementary Fig. 6A, B), and we therefore selected HCC1806 to test whether downregulation of *CCNE1* could rescue the sensitivity to the cell cycle checkpoint inhibitors. Two doxycycline-inducible shRNAs targeting *CCNE1* were transduced in these cells, and knockdown efficiency was assessed after 48 h of doxycycline treatment (Fig. 5a). Whereas sh*CCNE1*#1 showed a near-complete depletion of Cyclin E1, sh*CCNE1*#2 reproducibly resulted in a partial yet homogeneous knock-down throughout the cell population (Fig. 5a–c). In line with Cyclin E1 being a driver oncogene on the 19q12 amplicon, cell cycle analysis demonstrated that severe depletion of Cyclin E1 levels using sh*CCNE1*#1 in HCC1806 cells reduced the percentage of cells in S-phase (Fig. 5d), which was accompanied by a near-complete loss of clonogenic potential (Fig. 5e–g). In contrast, partial reduction of Cyclin E1 expression using sh*CCNE1*#2 cells did not significantly reduce the fraction of S-phase cells, nor did it compromise clonogenic potential or colony size (Fig. 5d–g).

To evaluate the effects of Cyclin E1 downregulation on replication kinetics, we analyzed DNA fibers of HCC1806 cells (Fig. 5h). Interestingly, knockdown of Cyclin E1 cells resulted in increased DNA synthesis speed in HCC1806 cells, as judged by IdU tract length (Fig. 5i). In addition, flow cytometry analyses demonstrated a reduction of intensity of the DNA damage and replication stress marker γH2AX upon Cyclin E1 knock-down (Fig. 5j). We next tested whether the observed reduction of replication stress levels in the Cyclin E1 knock-down cells also resulted in a reduction of mitotic aberrancies. Of note, the base-line frequency of mitotic errors in untreated HCC1806-sh*Luc* cells was ~50% (Fig. 5k), which is 10-fold higher than in non-transformed RPE-1-*TP53*^wt^ cells (Fig. 1e). Partial depletion of Cyclin E1 resulted in a dramatic reduction of mitotic errors to ~20% (Fig. 5k). Live-cell microscopy demonstrated that while mitotic duration was similar in all conditions (Fig. 5l), the percentage of mitotic errors is reduced ~1.5-fold (Fig. 5m). Combined, our data show that reducing Cyclin E1 expression levels in a Cyclin E1-overexpressing TNBC model, reduces replication stress levels and mitotic errors.

### ATR and WEE1 inhibitor sensitivity in cyclin E1-overexpressing cells

**Fig. 6:**
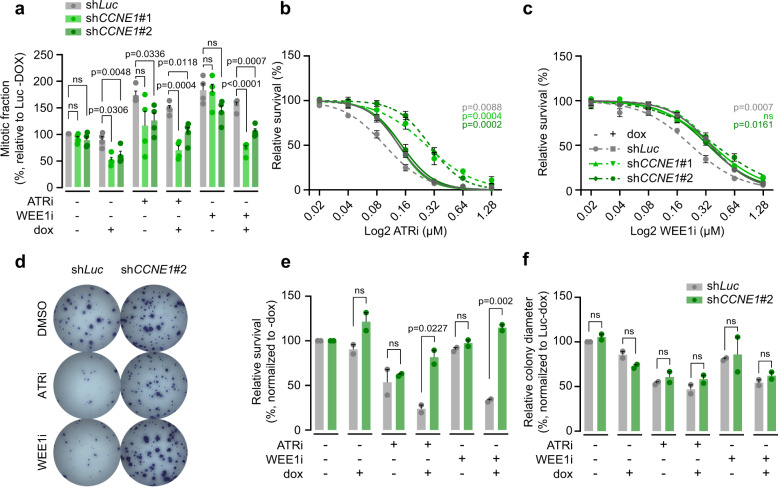
Cyclin E1 overexpression is required for ATR and WEE1 inhibitor sensitivity. **a** HCC1806 cell lines were induced to express Cyclin E1 shRNA for 2 days and were then treated with 0.25 µM of ATR inhibitor (ATRi, VE-822) or 0.1 µM of WEE1 inhibitor (WEE1i, MK-1775) for 8 h. Cells were then fixed and stained for DNA content (propidium iodine) and for mitotic population (MPM2) and analyzed using flow cytometry. Bars represent the mean and standard error of the mean (SEM) mitotic fraction of four independent experiments, normalized to untreated Luc-dox; *p*-values were calculated using two-tailed Student’s *t*-test **b**, **c** HCC1806 cell lines were induced to express Cyclin E1 shRNA and were subsequently treated for 3 days with ATR inhibitor (ATRi, VE-822) (panel **b**) or WEE1 inhibitor (WEE1i, MK-1775) (panel **c**) in a range from 0 to 1.28 µM. Subsequently, relative cell survival was assessed using MTT conversion as a proxy. Averages and standard error of the means (SEM) of three biological replicates are plotted. Reported *p*-values were calculated by a Student’s *t*-test comparing the area under the curve of doxycycline untreated samples to the curve of the doxycycline-treated samples. **d** Doxycycline-inducible HCC1806 cells were plated in six-well plates and allowed to attach for 24 h. Subsequently, cells were treated with doxycycline and 0.05 µM of ATR inhibitor (ATRi, VE-822) or 0.08 µM of WEE1 inhibitor (WEE1i, MK-1775). After 11 days, surviving colonies were stained. **e** Quantification of clonogenic survival from panel **d**. Bars represent the mean and SEM of clonogenic survival, relative to the non-doxycycline treated controls of two independent experiments; *p*-values were calculated using two-tailed Student’s *t*-test. **f** Quantification of colony diameter, relative to non-treated shLuc cells of two independent experiments. Bars represent mean and SEM; *p*-values were calculated using two-tailed Student’s *t*-test.

We next investigated how downregulation of Cyclin E1 impacts on replication stress and ATR and WEE1 inhibitor sensitivity. ATR or WEE1 inhibition increased γH2AX intensity levels (Supplementary Fig. 6C, D), although levels of mitotic errors were not further increased, likely because of the high base-line levels of mitotic errors in the HCC1806 cell (Supplementary Fig. 6E). Importantly, partial Cyclin E1-depletion consistently lowered γH2AX intensities and mitotic aberrancies observed in ATR or WEE1 inhibitor-treated cells (Supplementary Fig. 6E). Moreover, treatment with ATR and WEE1 inhibitor increased the mitotic fraction of HCC1806 cells ~2-fold, which was completely rescued by depletion of Cyclin E1 (Fig. 6a). Moreover, we observed Cyclin E1 depletion to confer resistance to ATR or WEE1 inhibition (Fig. 6b, c). Similarly, partial Cyclin E1 knockdown using sh*CCNE1*#2 resulted in increased clonogenic survival of WEE1 inhibitor-treated HCC1806 cells (Fig. 6d–f). Combined, our data indicate that Cyclin E1 overexpression is not only sufficient to drive sensitivity to ATR and WEE1 inhibition, but is also required for these effects.

## Discussion

In this report, we investigated the effects of oncogene-induced replication stress on mitotic fidelity and on the sensitivity to cell cycle checkpoint kinase inhibitors. We demonstrated that overexpression of Cdc25A or Cyclin E1 resulted in severe replication stress, which was associated with the induction of chromatin bridges and lagging chromosomes during mitosis. Furthermore, we observed that oncogene-induced replication stress sensitized cells to ATR and WEE1 checkpoint kinase inhibitors. ATR and WEE1 inhibition exacerbated the mitotic aberrancies induced by Cyclin E1 or Cdc25A overexpression and increased cell death. Finally, we show downregulation of Cyclin E1 in TNBC cells to result in rescue of replication kinetics and reduced cytotoxicity of ATR and WEE1 inhibitors.

Our findings are in line with earlier reports in which ATR inhibitor sensitivity was associated with Cdc25A expression, and WEE1 inhibitor sensitivity was associated with Cyclin E expression^47,48^. Importantly, our data point towards a critical role for mitotic segregation defects in cell death following oncogene-induced replication stress. Furthermore, our data indicate that exacerbation of chromosome segregation defects during mitosis upon ATR and WEE1 inhibit is associated with cytotoxicity of these drugs in cells harboring oncogene-induced replication stress, which was previously reported for PARP inhibitors^32^.

A possible explanation for these observations is that acceleration of mitotic entry upon ATR and WEE1 inhibition, leaves cells with oncogene-induced replication stress with insufficient time to resolve replicative lesions. Subsequently, mitotic entry commences in the presence of severe DNA lesions, which precludes proper chromosome segregation and leads to cell death. Indeed, cells in which ATR or WEE1 inhibition induced mitotic chromosome segregation defects showed a proportional increase in inhibitor-induced cytotoxicity. Specifically, RPE-1 cells with Cdc25A overexpression showed more chromosomal segregation defects and sensitivity to ATR and WEE1 inhibition in both *TP53*^wt^ and *TP53*^−/−^ settings. Conversely, Cyclin E1-overexpressing cells were only sensitive to both agents when *TP53* was mutated. These observations are in good agreement with a role for p53 signaling in preventing genomic instability following Cyclin E1 amplification^6,18–20^.

An explanation for why Cdc25A-overexpressing cells are sensitive to ATR and WEE1 inhibitors in a *TP53* wild-type setting could lie in checkpoint abrogation resulting from Cdc25A overexpression^49^. Furthermore, whereas Cyclin E1 overexpression only leads to CDK2 activation, Cdc25A affects multiple CDKs, including CDK1^30^. As a consequence, Cdc25A amplification de-regulates both S-phase and G2/M progression^49^. Interestingly, our study demonstrates that WEE1 inhibition sensitizes tumor cells regardless of *TP53* mutations status. WEE1 inhibition was reported earlier to be primarily effective in *TP53* mutant cells^50^, which was attributed to a defective G1/S checkpoint in *TP53* mutant cells, leading to increased reliance on their G2/M checkpoint. However, recent reports have shown that *TP53* mutation status alone does not explain responses of tumors to WEE1 inhibition, which underscore that WEE1 inhibitor sensitivity is more complex and multifactorial^45,48,51^.

As overexpression of Cyclin E1 leads to replication stress, increased mitotic aberrancies, and sensitivity to inhibition of ATR or WEE1, we wondered whether normalization of Cyclin E1 levels in TNBC cells harboring *CCNE1* amplification reduced these effects^46^. We observed that downregulation of Cyclin E1 resulted in elevated DNA replication speed, and diminished cytotoxic effects of ATR or WEE1 inhibition. These findings are in line with previous observations that Cyclin E1 overexpression contributes to the increased origin initiation and collisions between the replication and transcription machineries, which negatively impact replication speed and lead to replication fork collapse^17,42^. Such lesions create a dependence on replication checkpoint signaling, and explain the sensitivity of Cyclin E1-overexpressing cells to ATR and WEE1 inhibitors^48,52^, as well as the reversal of ATR and WEE1 inhibitor sensitivity upon Cyclin E1 downregulation.

Our data supports the notion that expression of replication stress-inducing oncogenes could be used as criteria to select patients for treatment with replication checkpoint kinase inhibitors, including ATR and WEE1. To test their value as biomarkers, it would be insightful to test ATR and WEE1 sensitivity in tumors harboring amplifications of different replication stress-inducing oncogenes, including *CCNE1*^53^, which is being used in a clinical trial to select patients for WEE1 inhibitor treatment (clinicaltrials.gov identifier: NCT03253679). In this context, cancers that currently lack drug targets are of particular interest, as these are difficult to treat, including triple-negative breast cancer.

Taken together, this study reports that replication stress induced by overexpression of Cyclin E1 and Cdc25A results in the formation of lagging chromosomes and chromatin bridges, which is further exacerbated by inhibition of ATR or WEE1 kinases, and results in exacerbated tumor cell killing. Conversely, normalization of Cyclin E1 levels restores replication kinetics and reduces the cytotoxicity from inhibition of ATR or WEE1 kinases. These insights could therefore help to guide novel treatment strategies for targeting genomically instable tumors harboring oncogene amplifications.

## Materials and methods

### Cell lines

hTERT-immortalized human RPE-1, human embryonic kidney 293 (HEK293T), HCC1806, HCC1569, and MDA-MB-157 cell lines were obtained from the American Type Culture Collection (#CRL4000, #CRL3216, #CRL2335, #CRL2330, and #HTB24) and regularly checked for mycoplasma and authenticated using STR profiling. RPE-1, HEK293T, and MDA-MB-157 cells were cultured in Dulbecco’s minimum essential media (DMEM, Thermofisher), complemented with 10% (v/v) fetal calf serum (FCS), 1% penicillin, and 1% streptomycin (Gibco). HCC1806 and HCC1569 cells were maintained in Roswell Park Memorial Institute medium (RPMI, Thermofisher) complemented with 10% FCS and 1% penicillin/streptomycin. All cells were grown at 37 °C in 20% O_2_ and 5% CO_2_ in a humidified incubator.

### Mutagenesis

CRISPR/Cas9 was used to mutate *TP53* in RPE-1 cells. To this end, a single guide RNA (sgRNA) (5′-CTGTCATCTTCTGTCCCTTC-3′) targeting exon 4 was cloned into pSpCas9(BB)-2A-GFP, which was provided by Feng Zhang (PX458, plasmid #48138, Addgene)^54^. Next, RPE-1 cells were transfected with PX458 and selected with Nutlin-3a (Axon Medchem, 10 μM) for 3 weeks. The viable cells were sorted into monoclonal lines using a MoFLO XDP or Sony cell sorter. *TP53* mutations in exon 4 were confirmed by Sanger sequencing and lack of p53 expression was confirmed by Western blot analysis. The reading frame of *TP53* was shifted by a 7 basepair deletion and a +217 bp insertion in Clone#1 and a −1 deletion and a +2 insertion in Clone#2 (Fig. 2a).

### DNA cloning and retroviral infections

RPE-1-*TP53*^wt^ and RPE-1-*TP53*^mut^ cell lines were engineered to express Cdc25A or Cyclin E1 in a doxycycline-dependent manner. To this end, human *CDC25A* was PCR amplified from FLAG-CDC25A-WT, which was a gift from Peter Stambrook^55^, using the following oligos: forward: 5′-CGCGGCCGCCATGGAACTGGGCCCGGAGCCC-3′, reverse: 5′-GATGAATTCTCACAGCTTCTTCAGACG-3′. Human *CCNE1* was PCR amplified from Rc-CycE, which was a gift from Bob Weinberg (Plasmid #8963, Addgene)^56^, using the following oligos: forward: 5′-CGCGGCCGCCATGAAGGAGGACGGCGGCGCG-3′, reverse: 5′-GATGAATTCTCACGCCATTTCCGGCCC-3′. The resulting fragments were cloned into pJET1.2/blunt, GeneJET (ThermoFisher). *CDC25A* and *CCNE1* were subcloned into pRetroX-Tight-Pur using NotI and EcoRI restriction sites. Subsequently, cell lines harboring pRetroX-Tet-On Advanced were transduced with pRetroX-Tight-Pur containing *CDC25A*, *CCNE1*, or empty plasmid. For transduction, HEK293T cells were transfected with 10 µg of pRetroX-Tet-On Advanced, 2.5 µg of pMDg, and 7.5 µg of pMDg/p as described previously^57^. After transduction, RPE-1 cell lines were selected for 7 days using geneticin (G418 Sulfate, 800 µg/mL, Thermofisher). Next, cell lines harboring pRetroX-Tet-On Advanced were transduced with pRetroX-Tight-Pur vectors containing *CDC25A* or *CCNE1*, and selected for 2 days with puromycin dihydrochloride (5 µg/mL, Sigma). To obtain cells stably expressing Histone H2B-EGFP, indicated RPE-1 cell lines were transduced as previously described^32^.

### RNA interference

For identifying endogenous Cyclin E1 on immunoblots, a SMARTpool siRNA mix (Dharmacon, Horizon Inspired Cell Solutions) for *CCNE1* was transfected at a final concentration of 80 nM with Oligofectamine (Invitrogen) according to the manufacturer’s instructions. To down-regulate *CCNE1* in HCC1806 cells, lentiviral shRNA interference sequences were clones into the Tet-pLKO-puro plasmid (a gift from Dimitri Wiederschain, #21915, Addgene^58^), following the depositor’s protocol. sh*CCNE1*#1 was designed to target exon 8 (5′-GCTTGTTCAGGAGATGAAATT-3′) and sh*CCNE1*#2 (sh#2) was designed to target exon 7 (5′-CGGTATATGGCGACACAAGAA-3′). A control shRNA-targeting luciferase (5′- AGAGCTGTTTCTGAGGAGCC-3′) was included in the experiments.

### Western blotting

After pretreatment with doxycycline, ATR inhibitor VE-822 (Axon), WEE1 inhibitor MK1775 (Axon MedChem), or Hydroxyurea (Sigma) at the indicated doses, cells were washed in PBS and lysed in MPER lysis buffer (Pierce), complemented with protease and phosphatase inhibitor cocktail (Thermo Scientific). Protein concentration was quantified using the Pierce BCA Protein Quantification Kit (Thermo Scientific). Lysates were resolved by SDS–polyacrylamide gels and transferred to polyvinylidene fluoride (PVDF) membranes (Immobilon). Membranes were incubated overnight at 4 °C with primary antibodies in Tris-buffered saline (Tris) containing 0.05% Tween-20 (Sigma) with 5% skimmed milk (Sigma). The following primary antibodies were used for Western blot analysis: mouse anti-Cdc25A (Santa Cruz Biotechnology, Sc-7389, 1:500), mouse anti-Cyclin E1 ([HE12], Abcam, ab3927, 1:1000), mouse anti-p53 ([DO-1], Santa Cruz Biotechnology, Sc-126, 1:1000), rabbit-anti-vinculin ([EPR8185], Abcam, ab129002, 1:2500), rabbit-anti-phospho ATM/ATR (Thr1989) Merck Millipore ABE462, 1:500), rabbit-anti-Phospho-Chk1 (Ser345) ([133D3], Cell Signaling, #2348, 1:500), Rabbit anti-phospho RPA32 (S33) (Bethyl Laboratories 1:1000), rabbit-anti-Phospho-Histone H2AX (Ser139) ([20E3], Cell Signaling, #9718, 1:1000), rabbit-anti-Recombinant Anti-CDK1 + CDK2 + CDK3 + CDK5 (phospho Y15) ([EPR7875], Abcam, ab133463, 1:1000) and mouse anti-beta-actin (MpBiomedicals, 69100, 1:10,000). Subsequently, membranes were incubated with corresponding horseradish peroxidase-conjugated secondary antibodies (1:2000, DAKO), and visualized with Lumi-Light (Roche Diagnostics). Images were captured with the ChemiDoc MP imaging system (Bio-Rad), and analyzed with the analyze gel module of the FIJI software.

### Flow cytometry

Flow cytometry was performed as described in ref. ^44^. Cells were stained with MPM2 antibody (Merck Millipore, 05-368, 1:000) and anti-γH2AX (Cell Signaling, #9718, 1:200), in combination with Alexa-488-conjugated and Alexa-647-conjugated secondary antibodies (1:200).

### Single-cell whole-genome analysis

RPE-1*-TP53*^-wt^ cells and RPE-1*-TP53*^−/−^ cell lines harboring doxycycline-inducible Cdc25A or Cyclin E1 were treated with doxycycline (1 µg/ml) for 120 h. Single-cell sequencing was performed as described in refs. ^43,44^.

### MTT assays

RPE-1-*TP53*^wt^ or RPE-1*-TP53*^−/−^ cell lines harboring doxycycline-inducible Cdc25A or Cyclin E1 were left untreated or treated with doxycycline (1 µg/ml) for 48 h. Subsequently, cells were re-plated in 96-wells at 10,000 cells per well in the continued presence or absence of doxycycline, and allowed to attach for 24 h. ATR inhibitor VE-822 (Axon) or WEE1 inhibitor MK-1775 (Axon MedChem) was added at indicated concentrations for 3 days. Next, cells were incubated with methylthiazol tetrazolium (MTT, final concentration 0.5 mg/ml) for 4 h. After removal of medium, formazan crystals were dissolved in dimethyl sulfoxide (DMSO). Absorbance was measured at 520 nm, and was quantified using a Benchmark III spectrophotometer (Bio-Rad). MTT conversion was plotted relative to the untreated cells. Per experiment, six technical replicates per condition were included. Averages and standard error of the means (SEM) of three biological replicates are plotted. The area under the curve was determined by Graphpad Prism 8 and used to test for statistical significance using a Student’s *t*-test.

### Live-cell microscopy

RPE-1-*TP53*^wt^ or RPE-1*-TP53*^−/−^ cell lines harboring doxycycline-inducible Cdc25A or Cyclin E1, transduced with H2B-EGFP were seeded in eight-chambered cover glass plates (Lab-Tek-II, Nunc). Cells were left untreated or treated with doxycycline (1 µg/ml) for 24 h, and were subsequently imaged for 48 h under the same treatment on a Delta Vision Elite microscope (×20 objective with 0.75 NA). Every 7 min, 10–15 images in the *Z*-plane were acquired with an interval of 0.5 µm. Mitotic entry was defined by NEB, and mitotic duration was defined as time between NEB and anaphase entry. Image analysis was done with SoftWorX software (Applied Precision/GE Healthcare).

Detailed descriptions of the following techniques are available in the Supplemental methods.

- DNA fiber analysis
- Immunofluorescence microscopy
- Flow cytometry
- Single-cell whole-genome analysis
- Clonogenic survival assays
- TCGA data set and CNA burden

## Supplementary information

Supplementary Methods

Supplemental Figure Legends

Supplementary Figure 1: Related to figure 1

Supplementary Figure 2: Related to figure 2

Supplementary Figure 3: mRNA expression of Cyclin E1 and Cdc25A are correlated with copy number alterations in various tumor types

Supplementary Figure 4: Cyclin E1 or Cdc25A overexpression induces genomic instability, related to figure 3.

Supplementary Figure 5: ATR or WEE1 inhibition do not affect ultra-fine bridge formation or mitotic timing, related to figure 4

Supplementary Figure 6: ATR and WEE1 inhibitor sensitivity in triple-negative breast cancer cells, related to figure 5

change of authorship agreement

## Data Availability

All sequencing data have been deposited at the European nucleotide archive under accession no. PRJEB32207.
