# Supplementary material for: Overexpression of Cyclin E1 or Cdc25A leads to replication stress, mitotic aberrancies, and increased sensitivity to replication checkpoint inhibitors
Source: Oncogenesis. 2020 Oct 7;9(10):88. doi: 10.1038/s41389-020-00270-2 (PMC7542455; doi:10.1038/s41389-020-00270-2)
Supplement: Supplementary file 1 — Supplementary Methods [file 41389_2020_270_MOESM1_ESM.docx]

## Supplemental methods

## DNA fiber analysis

RPE-1-*TP53*^wt^ or RPE-1*-TP53*^-/-^ cell lines harboring doxycycline-inducible Cdc25A and Cyclin E1 were pre-treated with doxycycline (1 µg/ml) for 48 hours, and subsequently pulse-labeled with CldU (25 µM) for 20 minutes at 37 °C. Subsequently, cells were washed three times with pre-warmed medium and then pulse-labeled with IdU (250 µM) for 20 minutes at 37 °C. After labeling, cells were harvested by trypsinization and re-suspended in cold PBS. Next, 2 μl of cell suspension was lysed on a microscopy slide by addition of 8 μl lysis solution (0.5% sodium dodecyl sulfate, 200 mM Tris [pH 7.4], 50 mM EDTA). After 5 minutes of incubation at room temperature, DNA fibers were spread by tilting the microscope slide, and were subsequently air-dried and fixed in methanol/acetic acid (3:1) for 10 minutes. Slides were washed twice in PBS, and DNA was denatured in 2.5M HCl for 75 minutes. DNA fibers were incubated in blocking solution (5% BSA in PBS) for 30 minutes, prior to incubation in primary antibodies (rat anti-BrdU, 1:1000, Abcam, ab6326; mouse anti-BrdU, 1:250, BD Biosciences, Clone B44) for 60 minutes at room temperature. After three washing steps in blocking solution, slides were incubated with secondary antibodies (Alexa488-conjugated anti-rat and Alexa594 or 647-conjugated anti-mouse, 1:500) for 1 hour at room temperature. Images were acquired on a Leica DM-6000B (63x immersion objective with 1.30 NA) fluorescence microscope, equipped with Leica Application Suite software. Per condition, the lengths of at least 250 IdU tracts were measured using ImageJ software. Statistical analysis was performed using the non-parametric Mann-Whitney U test with GraphPad Prism version 8.

## Immunofluorescence microscopy

Indicated cells were seeded on glass coverslips in 6-well plates for 24 hours. Subsequently, cells were treated with doxycycline (1 µg/ml) for 48 hours. Then, cells were treated with MK-1775 (100 nM) or VE-822 (250 nM) for 8 hours if indicated, and were subsequently fixed in 4% formaldehyde in PBS. Following permeabilizing for 5 minutes (0.1% Triton X-100 in PBS), cells were incubated with blocking buffer (3% BSA and 0.05% tween in PBS), cells were incubated overnight with mouse anti-PICH (1:1000, Novus Biologics, NBP2-13969), mouse anti-γH2AX (1:400, Millipore, 05-636) or mouse anti-Cyclin E1 ([HE12], Abcam, ab3927, 1:1000), and were then treated with Alexa-488 or Alexa-647-conjugated secondary antibodies and counterstained with DAPI. Images were acquired on a Leica DM6000B microscope using a 63x immersion objective (PL S-APO, numerical aperture: 1.30) with LAS-AF software (Leica). Using ImageJ software, Cyclin E1 staining intensity was measured in the nuclei which were selected based on the DAPI channel by the ‘Analyze Particle’ tool.

***Flow cytometry***

Cells where either analyzed as asynchronous cultures or were synchronized at the G1/S cell cycle transition using a double-thymidine block. Specifically, cells were treated with thymidine (2mM, Sigma) for 17 hours, washed twice with pre-warmed PBS, and were incubated in pre-warmed warm medium for 9 hours. Subsequently, cells were again incubated in thymidine for 17 hours, after which cells were washed with PBS and released in pre-warmed medium containing VE-822 (0.250 µM) or MK-1775 (0.1 µM), and harvested at indicated time points. When indicated, cells were trapped in mitosis using an 8-hour incubation with nocodazole (250 ng/ml, Sigma). Cells were then fixed in ice-cold ethanol (70%) for at least 16 hours and stained with MPM2 antibody (Merck Millipore, 05-368, 1:000) and anti-γH2AX (Cell Signaling, #9718, 1:200), in combination with Alexa-488-conjugated and Alexa-647-conjugated secondary antibodies (1:200). DNA staining was performed using propidium iodide in the presence of RNAse. For S-phase analysis, prior to fixation asynchronous cells were incubated with 10 µM of EdU (Invitrogen) for 45 minutes. Cells were permeabilized with 0.5% Triton-X-100 for 30 minutes and washed with 3% BSA-PBS. EdU click reaction was performed at room temperature by incubation for 30 minutes with staining cocktail final dilution of 43mM Tris-HCl pH 7.5, 1.6 mM CuSO_4_·5H_2_O, 25 μM ATTO 488 Azide (ATTO-TEC GmbH) and 1 mM Ascorbic Acid. At least 10,000 events per sample were analyzed on a FACScalibur or LSR-II (Becton Dickinson). Data was analyzed using FlowJo software.

## Single-cell whole-genome analysis

RPE-1*-TP53*^-wt^ cells and RPE-1*-TP53*^-/-^ cell lines harboring doxycycline-inducible Cdc25A or Cyclin E1 were treated with doxycycline (1 µg/ml) for 120 hours. Subsequently, cells were lysed and the G1 population was single-cell sorted into 96-well plates (48 cells per sample) using a Hoechst/Propidium iodide double staining. To perform sample preparation and generate Illumina-based libraries, a Bravo automated liquid handling platform (Agilent Technologies) was employed, as described previously (1). The libraries were sequenced on a NextSeq 500 sequencer (Illumina) and analyzed using AneuFinder software as previously described (2). RPE-1-*TP53*^wt^-Empty or RPE-1-*TP53*^-/-^-Empty (negative control) cells were employed as a reference to determine the deviation from the modal copy number state per sample and per bin. The focal copy number alterations (CNAs) scores were obtained from the bins that deviated from the modal copy number of the negative control.

## Clonogenic survival assays

HCC1806 cells were seeded in 6-well plates (at approximately 232 or 500 cells per well) and allowed to adhere for 24 hours. Subsequently, cells were treated with doxycycline (1 μg per ml) in the presence or absence of VE-822 (0.05 µM, Axon MedChem) or MK-1775 (0.08 µM, Axon MedChem). After 11 or 14 days, cells were fixed in methanol and stained in staining buffer (50% methanol, 29.95% water, 20% Acetic acid, and 0.05% of Coomassie Brilliant Blue). Images of colonies were obtained using an EliSpot reader (Alpha Diagnostics International) with vSpot Spectrum software. The number and size of colonies were measured using ImageJ software. Statistical analysis was performed using the non-parametric Mann-Whitney U test with GraphPad Prism 6.

***TCGA data set and CNA burden***

From TCGA, we obtained the pre-processed and normalized level 3 RNA-seq (version 2) data for 34 cancer datasets available at the Broad GDAC Firehose portal (downloaded January 2017 https://gdac.broadinstitute.org/). For each sample, we downloaded RNA-Seq with Expectation Maximization (RSEM) gene normalized data (identifier: illuminahiseq_rnaseqv2-RSEM_genes_normalized) (3). RNA-Seq expression level read counts were normalized using FPKM-UQ (Fragments per Kilo-base of transcript per Million mapped reads upper quartile normalization, NCI Genomic Data Commons (GDC), n.d.). The RNA-Seq expression level read counts for each of the samples were log2-transformed. Publicly available inferred CNA burden data for TCGA were obtained from http://www.genomicinstability .org/ (4). The association between CNA burden and mRNA expression of CCNE1, CDC25A for the TCGA-dataset were quantified using Spearman correlation coefficient. This analysis was conducted separately for different cancer types.

1. van den Bos H, Spierings DCJ, Taudt AS, Bakker B, Porubský D, Falconer E, et al. Single-cell whole genome sequencing reveals no evidence for common aneuploidy in normal and Alzheimer’s disease neurons. Genome Biol. 2016 May;17(1):116.

2. Bakker B, Taudt A, Belderbos ME, Porubsky D, Spierings DCJ, de Jong T V, et al. Single-cell sequencing reveals karyotype heterogeneity in murine and human malignancies. Genome Biol. 2016 May;17(1):115.

3. Li B, Dewey CN. RSEM: accurate transcript quantification from RNA-Seq data with or without a reference genome. BMC Bioinformatics [Internet]. 2011;12(1):323. Available from: https://doi.org/10.1186/1471-2105-12-323

4. Bhattacharya A, Bense RD, Urzúa-Traslaviña CG, de Vries EGE, van Vugt MATM, Fehrmann RSN. Transcriptional effects of copy number alterations in a large set of human cancers. Nat Commun. 2020 Feb;11(1):715.
