# Supplementary material for: Overexpression of Cyclin E1 or Cdc25A leads to replication stress, mitotic aberrancies, and increased sensitivity to replication checkpoint inhibitors
Source: Oncogenesis. 2020 Oct 7;9(10):88. doi: 10.1038/s41389-020-00270-2 (PMC7542455; doi:10.1038/s41389-020-00270-2)
Supplement: Supplementary file 2 — Supplemental Figure Legends [file 41389_2020_270_MOESM2_ESM.docx]

**Supplemental Figure Legends**

**Supplementary Figure 1: Related to figure 1**

**a** TGCA analysis of Cyclin E1 and Cdc25A mRNA expression in pan cancer, breast cancer (ER+/HER2-), and TNBC breast cancer**. b** Longer exposure of Cyclin E1 immunoblot presented in Fig 1a in RPE-1-*TP53*^wt^ cell lines induced to express Cyclin E1 or Cdc25A. Black arrow indicates an aspecific band, red arrow indicates endogenous Cyclin E1. **c** RPE-1-*TP53*^wt^ were treated for 48 hours with doxycycline to induce expression of Cyclin E1 or CDC25A. **c** The percentages of anaphase or telophase cells containing ultra-fine bridges (n=3, *n*>25 per experiment) were quantified. *p-*values were calculated using two-tailed Student’s t-test.

**Supplementary Figure 2: Related to figure 2**

**a** Longer exposure of Cyclin E1 immunoblot presented in Fig 2B in RPE-1-*TP53*^-/-^ cl#1 cell lines induced to express Cyclin E1 or Cdc25A. Black arrow indicates an aspecific band, red arrow indicates endogenous Cyclin E1. **b** RPE-1-*TP53*^-/-^ cl#2 cells were engineered to overexpress empty, Cyclin E1 or Cdc25A constructs in a doxycycline-inducible manner. Immunoblot shows Cyclin E1, Cdc25A, p53 and Vinculin protein levels at 48 hours after addition of doxycycline (dox). RPE-1-*TP53*^wt^ cells were used as a positive control for p53. **c** RPE-1-*TP53*^wt^, RPE-1-*TP53*^-/-^ cl#1 and RPE-1-*TP53*^-/-^ cl#2 were treated as in b. Immunoblot shows Cyclin E1, Cdc25A, p53 and Vinculin protein levels at 48 hours after addition of doxycycline (dox). **d** Cells were treated as in b and subsequently labeled for 20 minutes with CldU (25 µM) and for 20 minutes with IdU (250 µM). At least 473 were analyzed. Graphs show individual data points, median and interquartile range. *p*-values were calculated using the Mann-Whitney U test**. e** RPE-1-*TP53*^-/-^ cl#2 were treated for 48 hours with doxycycline to induce expression of Cyclin E1 or Cdc25A. Cells were stained with α-Tubulin (red) and counterstained with DAPI (blue). Quantification of anaphase and telophase cells containing chromatin bridges and/or lagging chromosomes. The bars represent the mean and standard error or the mean (SEM) from 3 experiments, n>25 per experimental condition; *p*-values were calculated using two-tailed Student’s t-test. **f** RPE-1-*TP53*^wt^ were treated as described in e. Cells were stained for PICH and α-Tubulin and counterstained with DAPI. The percentages of anaphase or telophase cells containing ultra-fine bridges were quantified. The bars represent the mean and standard error or the mean (SEM) from 3 experiments *n*>25 per experimental condition; *p-*values were calculated using two-tailed Student’s t-test.

**Supplementary Figure 3: mRNA expression of Cyclin E1 and Cdc25A are correlated with copy number alterations in various tumor types**

**a,b** CCNE1 or Cdc25A read count were correlated to copy number load using Spearman’s correlation in panel a triple negative breast cancer and in panel b ovarian carcinomas. **c** Spearman’s correlation coefficient was calculated between copy number load and read counts of CCNE1 or Cdc25A for patient samples from various tumor types in TCGA data.

**Supplementary Figure 4: Cyclin E1 or Cdc25A overexpression induces genomic instability, related to figure 3.**

**a** RPE-1-*TP53*^wt^ (n=44), RPE1-*TP53*^-/-^ #cl1 (n=47)**,** RPE1-*TP53*^-/-^#cl2 (n=42) were treated with doxycycline for 120 hours. After single cell sorting, genomic DNA was harvested for single-cell whole Genome Sequencing (sc-WGS). Genome-wide copy number plots were generated using AneuFinder software. Each panel displays the individual cells in rows, and the chromosomes numbers from 1-X in columns. Absolute copy number states for each cell are depicted in different colors. **b** Genome-wide copy number deviation plots of RPE-*TP53*^-/-^ cl#2 empty (n=42)**,** RPE-*TP53*^-/-^ cl#1-Cyclin E1 (n=48) and RPE-*TP53*^-/-^ cl#1**-**Cdc25A cells (n=43). Cells were treated as in a. The modal copy number state is pictured in green, deviations of the modal copy number state, both focal and whole-chromosome, are colored red. **c** Copy-number alterations (CNAs) per cell were calculated according to the modal state. Medians with interquartile range are depicted and statistical analyses were performed using a One-sided Mann Whitney U test. **d** Whole numerical chromosomes per cell were counter per single cell. Medians with interquartile range are depicted and statistical analyses were performed using a One-sided Mann Whitney U test.

**Supplementary Figure 5: ATR or WEE1 inhibition do not affect ultra-fine bridge formation or mitotic timing, related to figure 4**

**a,b** RPE-*TP53*^wt^ (a) and RPE-*TP53*^-/-^ cl#1 (b) cells were treated as described in Fig. 4a, and immunoblotted for Cyclin E and Cdc25A overexpression. Vinculin serves as a loading control. **c** RPE-1-*TP53*^wt^ cells induced to express Cdc25A or Cyclin E1 were treated with ATR inhibitor (VE-822, 0.25 µM) or WEE1 inhibitor (MK-1775, 0.1 µM) for 8 hours if indicated. The percentages of anaphase or telophase cells containing chromatin bridges or lagging chromosomes were quantified. The bars represent mean and SEM from 3 experiments, *n*>30 per experimental condition; The p-values were calculated by one-way ANOVA (P<0.0001) and followed by Sidak’s Multiple Comparison Test. **d** RPE-1-*TP53*^wt^ were treated as described in c. cells were stained for PICH and α-Tubulin and counterstained with DAPI. The percentages of anaphase or telophase cells containing ultra-fine bridges were quantified. The bars represent the mean and standard error or the mean (SEM) from 3 experiments *n*>25 per experimental condition; *p-*values were calculated using two-tailed Student’s t-test. **e** RPE-*TP53*^-/-^ cl#2 cells were treated as described in panel c. The percentages of anaphase or telophase cells containing ultra-fine bridges were quantified. The bars represent the mean and standard error or the mean (SEM) from 3 experiments *n*>25 per experimental condition; *p-*values were calculated using two-tailed Student’s t-test. **f** Duration of mitosis in RPE-1-*TP53*^-/-^ cl#1 cell lines harboring doxycycline-inducible Cdc25A or Cyclin E1, transduced with H2B-EGFP. Cells were pre-treated for 24 hours with doxycycline, after which cells were treated with 0.25 µM of ATR inhibitor (ATRi, VE-822) or 0.1µM of WEE1 inhibitor (WEE1i, MK-1775), and subsequently followed with live-cell microscopy for 48 hours using 7-minute intervals. Duration of mitosis was measured as the time between nuclear envelope breakdown (NEB) and anaphase entry. A Gaussion curve was fitted to the data, and a *p*-value was calculated using a Kruskal-Wallis multiple-comparison test. **g** RPE-*TP53*^wt^ were treated for 48 hours with doxycycline to induce expression of Cyclin E1 or CDC25A and subsequently incubated with thymidine (2mM) for 17 hours. Cells were then released for 9 hours in pre-warmed growth media and again treated for 17 hours with thymidine prior to release in growth media supplemented with DMSO, 0.25 µM of ATR inhibitor (ATRi, VE-822) or 0.1 µM of WEE1 inhibitor (WEE1i, MK-1775). Cells were then fixed and indicated time points and stained for DNA content (propidium iodine) and for MPM2 and analysed using flow cytometry a minimum of 20,000 events was analyzed per sample.

**Supplementary Figure 6: ATR and WEE1 inhibitor sensitivity in triple-negative breast cancer cells, related to figure 5**

**a, b** Triple-negative breast cancer cell lines MDA-MB157, HCC1569 and HCC1806 were treated for 3 days with ATR inhibitor VE-822 in a range from 0 µM to 3.2 µM (a), or WEE1 inhibitor (MK-1775) in a range from 0 µM to 1.28 µM (b) Subsequently, MTT conversion was analyzed. Averages and standard error of the means (SEM) of 3 biological replicates are plotted. **c** Example of flow cytometry plot measuring γH2AX intensity and DNA content in HCC1806 cells. **d** HCC1806 cell lines were induced to express Cyclin E1 shRNA for 2 days and were then treated with 0.25 µM of ATR inhibitor (ATRi, VE-822) or 0.1 µM of WEE1 inhibitor (WEE1i, MK-1775) for 8 hours. Cells were then fixed and stained for DNA content (propidium iodine) and for γH2AX and analyzed using flow cytometry a minimum of 20,000 events was analyzed per sample. Bars represent the mean and standard error of the mean (SEM) mitotic fraction of 3 independent experiments, normalized to untreated Luc – dox. DMSO-control cells are the same as in figure 5j. *p*-values were calculated using two-tailed Student’s t-test. **e** Cyclin E knock-down was induced by doxycycline for 48 hours. Cells were then fixed and the percentage of mitotic aberrancies was quantified. DMSO-control cells are the same as in figure 5k. At least 30 mitoses were analyzed for each experimental condition. Data represents mean and SEM of three independent experiments; The p-values were calculated by one-way ANOVA (P<0.0001) and followed by Sidak’s Multiple Comparison Test.
