# Supplementary figures and images for: Overexpression of Cyclin E1 or Cdc25A leads to replication stress, mitotic aberrancies, and increased sensitivity to replication checkpoint inhibitors
Source: Oncogenesis. 2020 Oct 7;9(10):88. doi: 10.1038/s41389-020-00270-2 (PMC7542455; doi:10.1038/s41389-020-00270-2)

Supplemental Figure 1

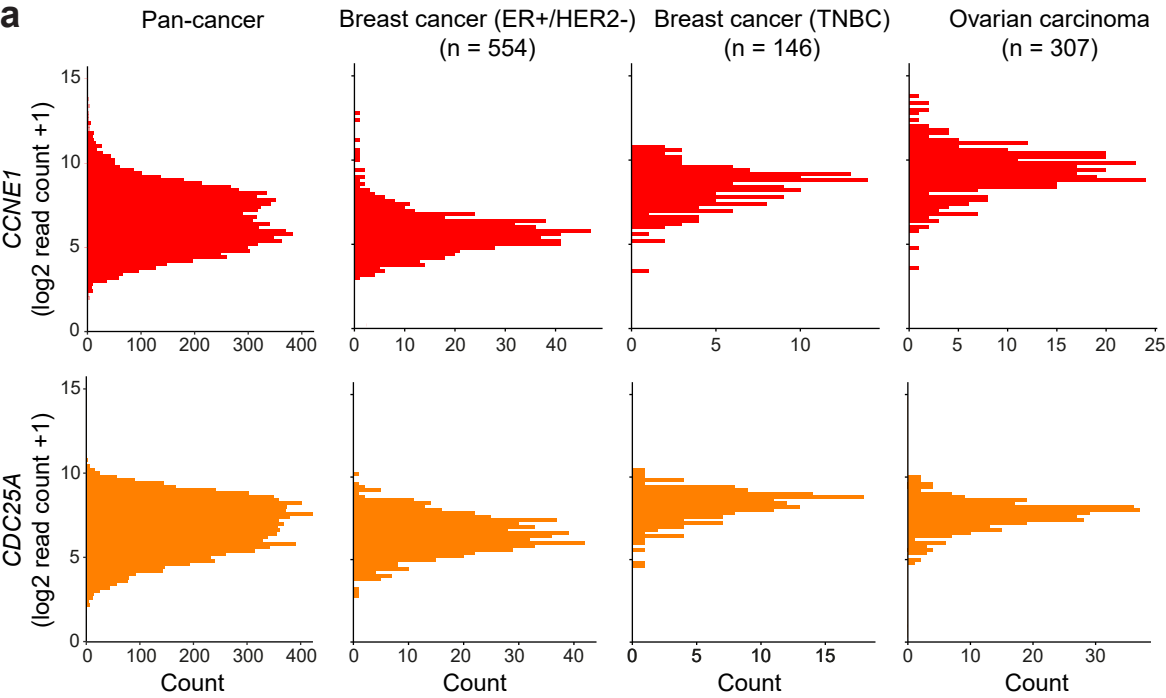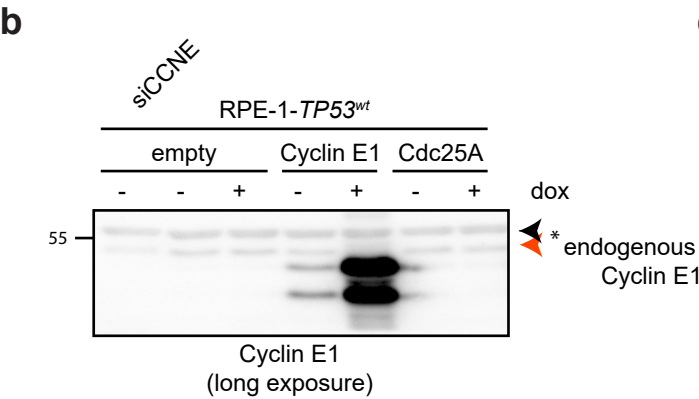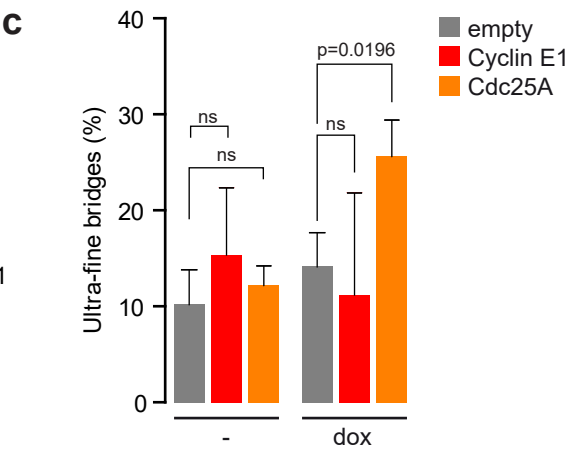

Supplement: Supplementary file 3 — Supplementary Figure 1: Related to figure 1 [file 41389_2020_270_MOESM3_ESM.pdf]

Supplemental Figure 2

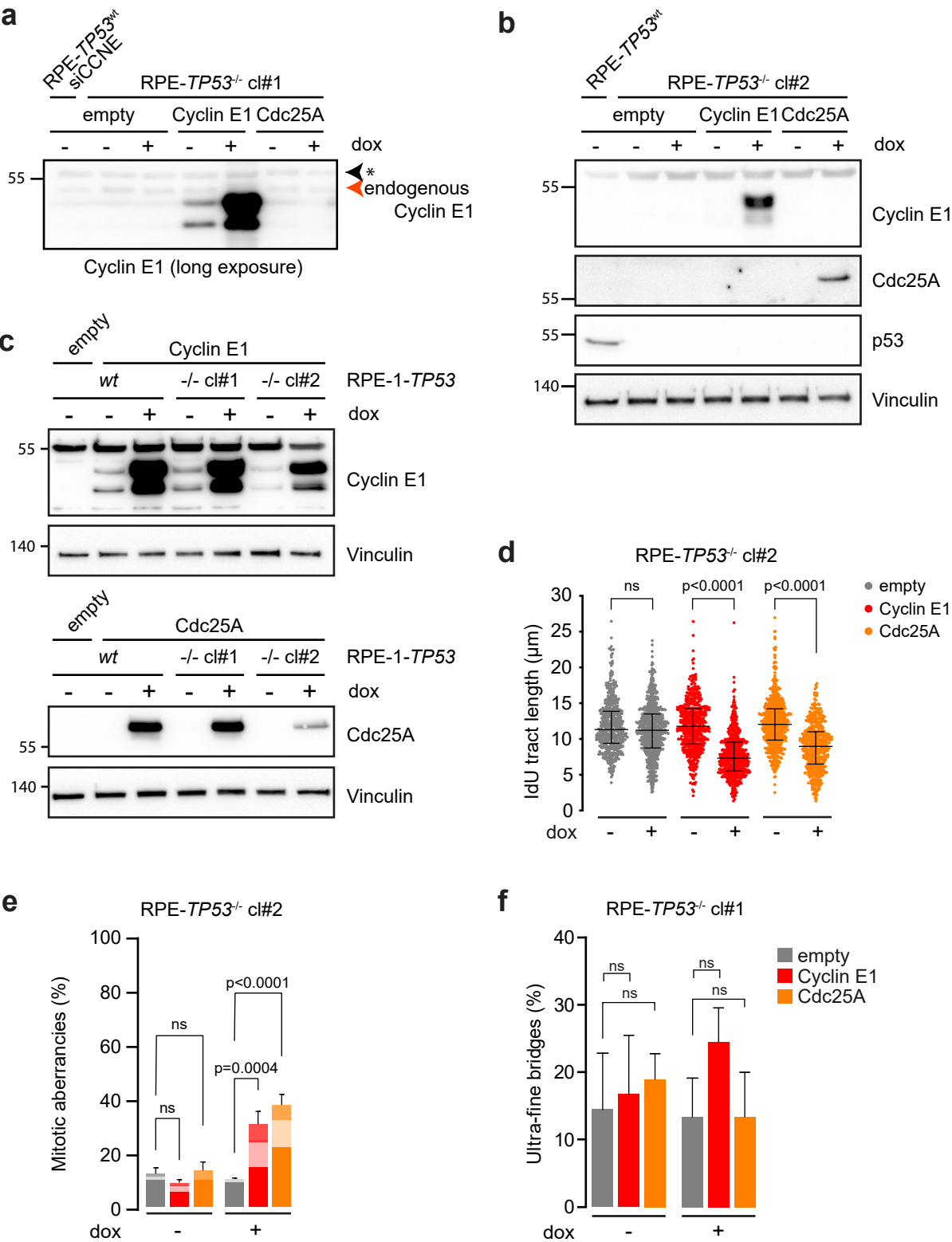

Supplement: Supplementary file 4 — Supplementary Figure 2: Related to figure 2 [file 41389_2020_270_MOESM4_ESM.pdf]

Supplemental Figure 3

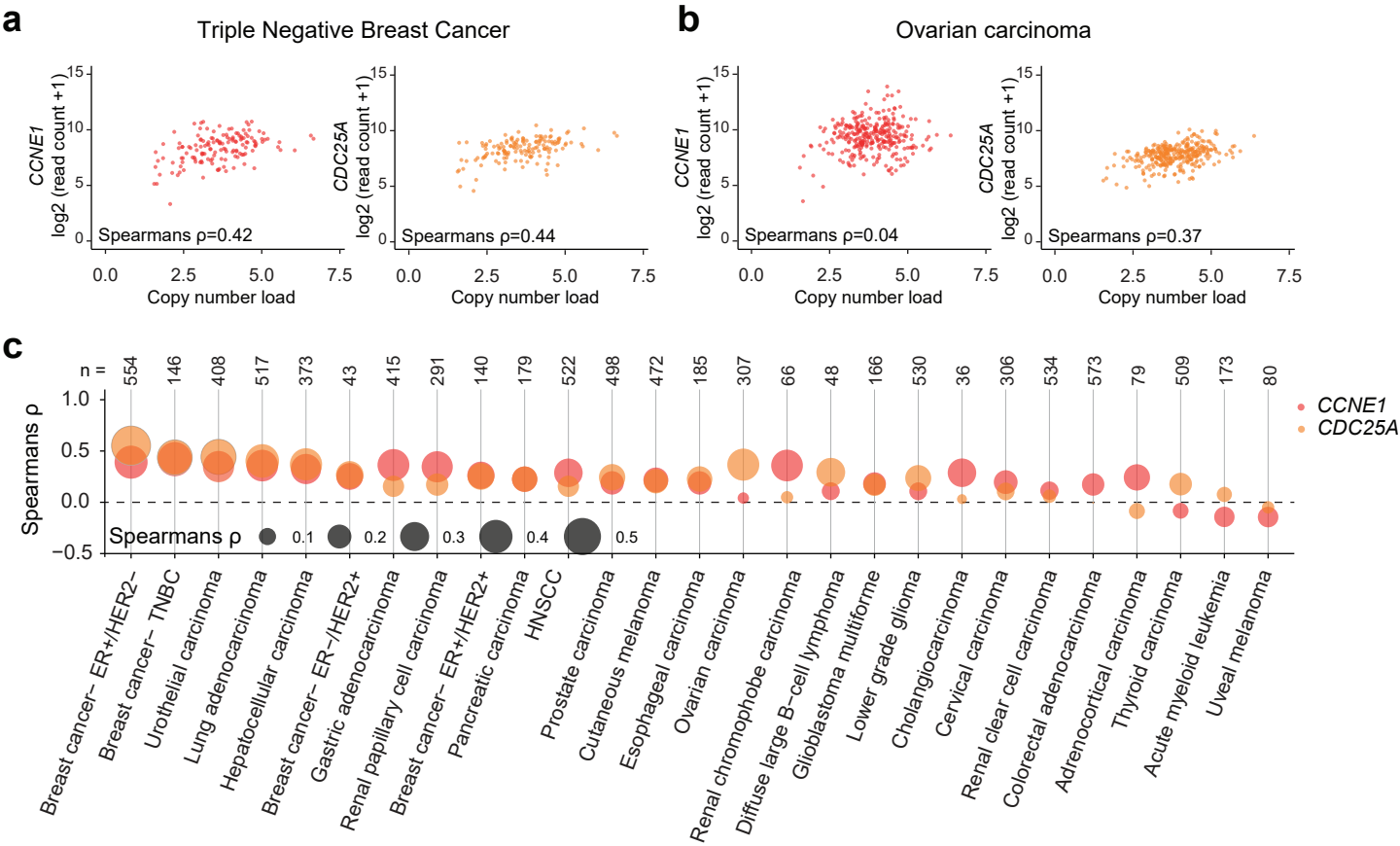

Supplement: Supplementary file 5 — Supplementary Figure 3: mRNA expression of Cyclin E1 and Cdc25A are correlated with copy number alterations in various tumor types [file 41389_2020_270_MOESM5_ESM.pdf]

# Supplemental Figure 4

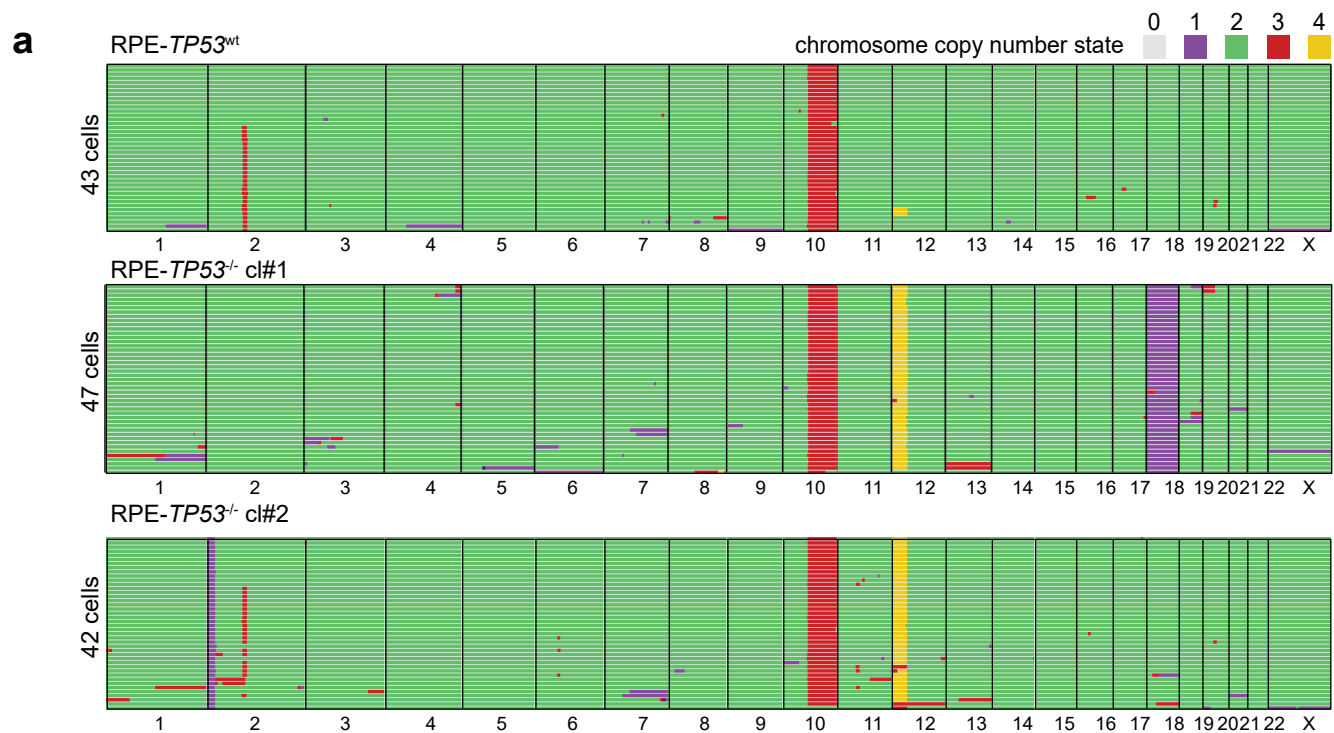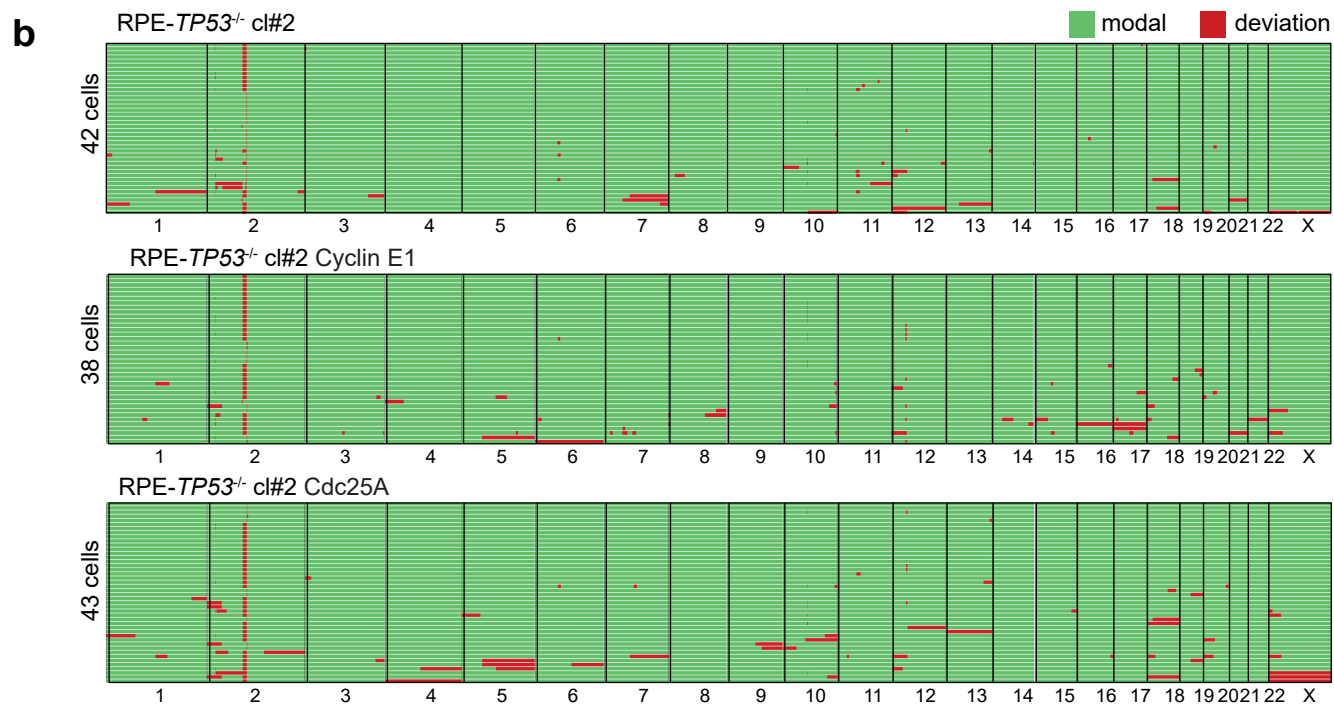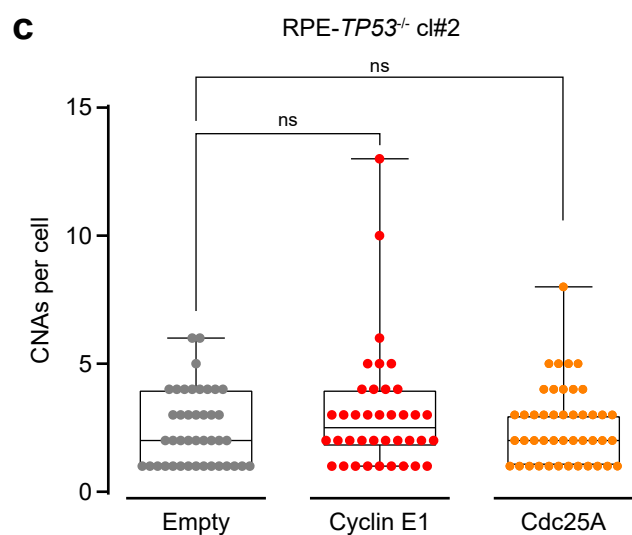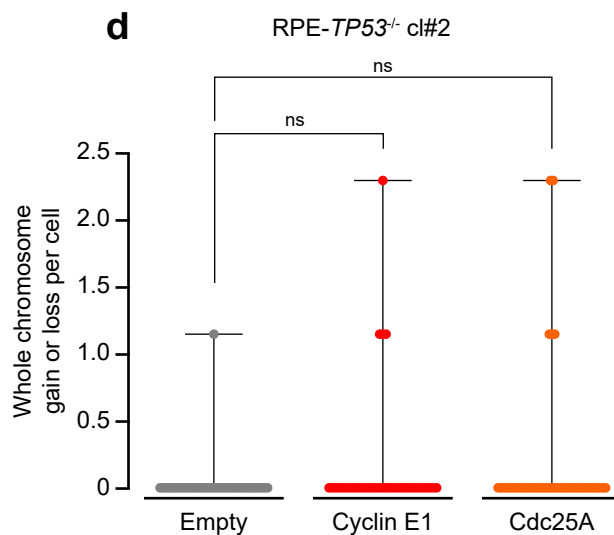

Supplement: Supplementary file 6 — Supplementary Figure 4: Cyclin E1 or Cdc25A overexpression induces genomic instability, related to figure 3. [file 41389_2020_270_MOESM6_ESM.pdf]

Supplemental Figure 5

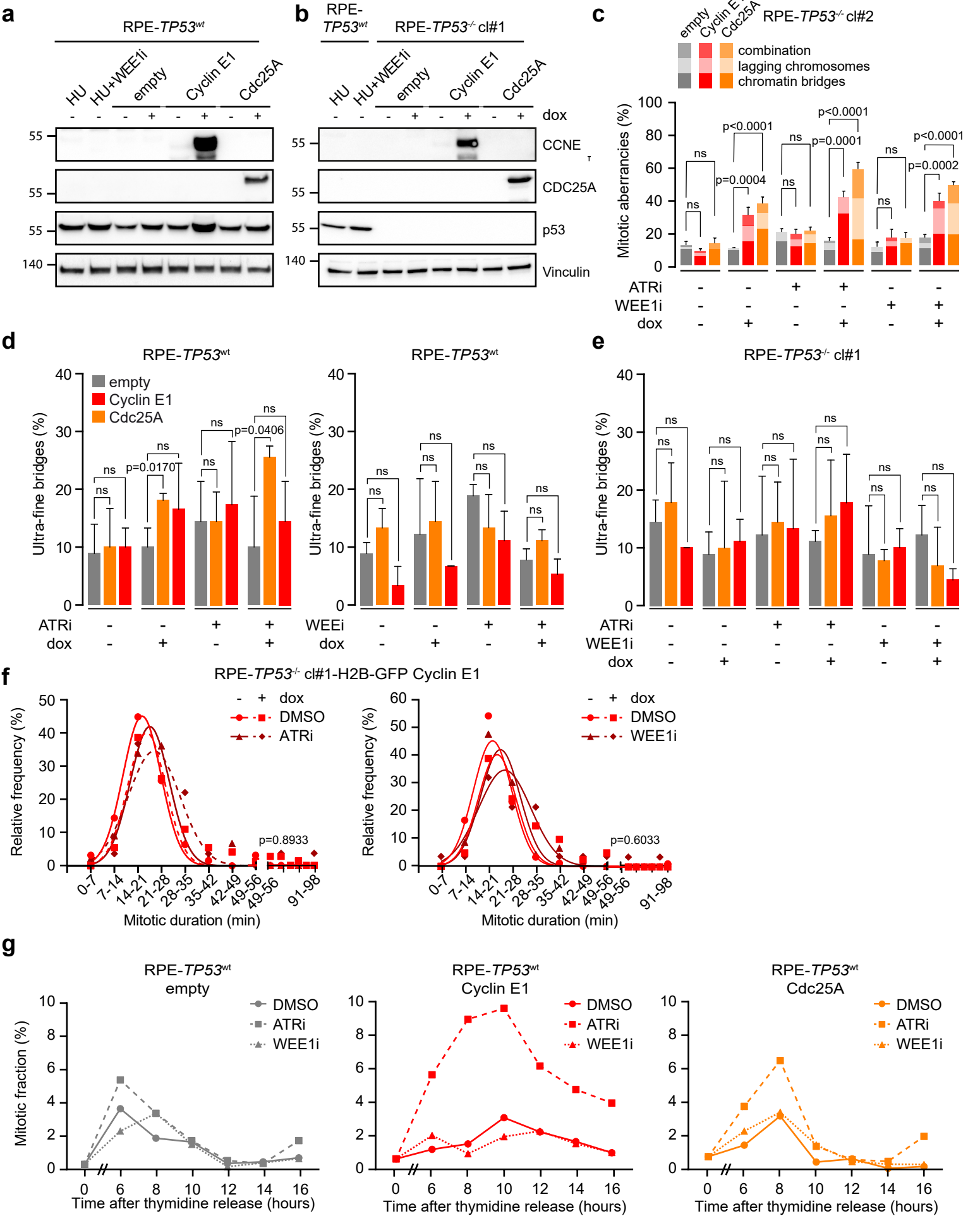

Supplement: Supplementary file 7 — Supplementary Figure 5: ATR or WEE1 inhibition do not affect ultra-fine bridge formation or mitotic timing, related to figure 4 [file 41389_2020_270_MOESM7_ESM.pdf]

Supplemental Figure 6

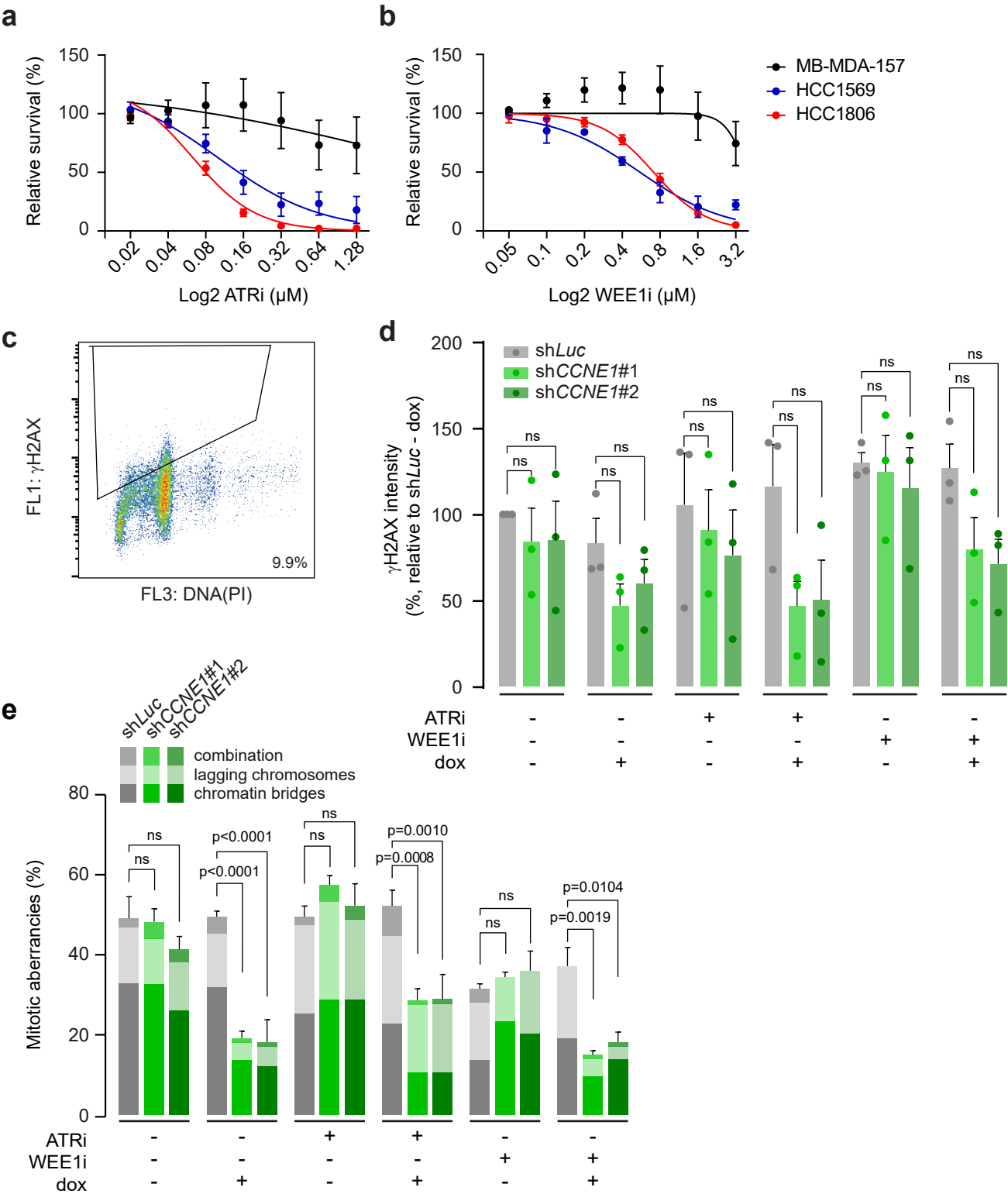

Supplement: Supplementary file 8 — Supplementary Figure 6: ATR and WEE1 inhibitor sensitivity in triple-negative breast cancer cells, related to figure 5 [file 41389_2020_270_MOESM8_ESM.pdf]
