# Supplementary material for: Overexpression of Cyclin E1 or Cdc25A leads to replication stress, mitotic aberrancies, and increased sensitivity to replication checkpoint inhibitors
Source: Oncogenesis. 2020 Oct 7;9(10):88. doi: 10.1038/s41389-020-00270-2 (PMC7542455; doi:10.1038/s41389-020-00270-2)
Supplement: Supplementary file 9 — change of authorship agreement [file 41389_2020_270_MOESM9_ESM.pdf]

From: **Kok, YP (onco)** y.p.kok@umcg.nl
Subject: RE: Authorship request....
Date: 9 September 2020 at 12:53
To: Vugt, MATM van m.vugt@umcg.nl, Sergi Guerrero sergibiotec@gmail.com, Schoonen, PM p.m.schoonen@umcg.nl, Everts, M evertsm@umcg.nl, Bhattacharya, A (onco) a.bhattacharya@umcg.nl, Fehrmann, RSN (int) r.s.n.fehrmann@umcg.nl , Tempel, N van den (onco) n.van.den.tempel@umcg.nl

---

I agree.

Kind regards,
Yannick Kok

From: Vugt, MATM van
Sent: woensdag 9 september 2020 12:02
To: Kok, YP (onco); Sergi Guerrero; Schoonen, PM; Everts, M; Bhattacharya, A (onco); Fehrmann, RSN (int); Tempel, N van den (onco)
Subject: Authorship request....

Dear all,

I just sent you an email with a request to agree with the new authorship. This is the exact same authorship change that I asked you to agree on before, but the old/new authorship needed to be included.

Could you please reply to that email by saying that you agree? Thanks for your help!

Marcel

Could you please reply to this email if you agree with the updated authorship for the manuscript 'Overexpression of Cyclin E1 or Cdc25A leads to replication stress, mitotic aberrancies and increased sensitivity to replication checkpoint inhibitors'.

Original authorship (with Sergi Guerrero Llobet and Pepijn Schoonen sharing first authorship):

Sergi Guerrero Llobet[1, *], Pepijn M. Schoonen[1, *], Marieke Everts[1], Yannick P. Kok[1], Nathalie van den Tempel[1], Marcel A.T.M. van Vugt[1,2]

New authorship (with Yannick Kok, Sergi Guerrero Llobet and Pepijn Schoonen sharing first authorship, and Nathalie van den Tempel and Marcel Van Vugt sharpen senior authorship and correspondence)?

Yannick P. Kok[1,*], Sergi Guerrero Llobet[1,*], Pepijn M. Schoonen[1,*], Marieke Everts[1], Arkajyoti Bhattacharya[1], Rudolf S.N. Fehrmann[1], Nathalie van den Tempel[1,*], Marcel A.T.M. van Vugt[1,*]

Kind regards, Marcel

Marcel A.T.M. van Vugt, PhD

[illegible]

Department of Medical Oncology
University Medical Center Groningen
PO box 30.001
Room F1.23/DA13
Hanzeplein 1
9700 RB Groningen
The Netherlands

T: +31(0)50-3615002
F: +31(0)50-3614862
E: m.vugt@umcg.nl

From: **Fehrmann, RSN (int)** r.s.n.fehrmann@umcg.nl
Subject: Re: Oncogenesis authorship
Date: 9 September 2020 at 12:02
To: Vugt, MATM van m.vugt@umcg.nl
Cc: Kok, YP (onco) y.p.kok@umcg.nl, Sergi Guerrero sergibiotec@gmail.com, Schoonen, PM p.m.schoonen@umcg.nl, Everts, M evertsm@umcg.nl, Bhattacharya, A (onco) a.bhattacharya@umcg.nl, Tempel, N van den (onco) n.van.den.tempel@umcg.nl

---

I agree.

Gr Rudolf

Rudolf S.N. Fehrmann, MD/PhD I internist/medical oncologist/principal investigator I Department of Medical Oncology I University Medical Center Groningen / Cancer Research Center Groningen I Address: postbox 30.001, 9700RB Groningen, The Netherlands I E-mail: r.s.n.fehrmann@umcg.nl I Website: http://www.rudolffehrmann.nl

> On 9 Sep 2020, at 11:59, Vugt, MATM van <m.vugt@umcg.nl> wrote:
>
> Dear co-authors,
>
> Could you please reply to this email if you agree with the updated authorship for the manuscript 'Overexpression of Cyclin E1 or Cdc25A leads to replication stress, mitotic aberrancies and increased sensitivity to replication checkpoint inhibitors'.
>
> Original authorship (with Sergi Guerrero Llobet and Pepijn Schoonen sharing first authorship):
>
> Sergi Guerrero Llobet[1, *], Pepijn M. Schoonen[1, *], Marieke Everts[1], Yannick P. Kok[1], Nathalie van den Tempel[1], Marcel A.T.M. van Vugt[1,2]
>
> New authorship (with Yannick Kok, Sergi Guerrero Llobet and Pepijn Schoonen sharing first authorship, and Nathalie van den Tempel and Marcel Van Vugt sharpen senior authorship and correspondence)?
>
> Yannick P. Kok[1,*], Sergi Guerrero Llobet[1,*], Pepijn M. Schoonen[1,*], Marieke Everts[1], Arkajyoti Bhattacharya[1], Rudolf S.N. Fehrmann[1], Nathalie van den Tempel[1,*,], Marcel A.T.M. van Vugt[1,*]
>
> Kind regards, Marcel
>
> --
>
> Marcel A.T.M. van Vugt, PhD
>
> Department of Medical Oncology
> University Medical Center Groningen
> PO box 30.001
> Room F1.23/DA13
> Hanzeplein 1
> 9700 RB Groningen
> The Netherlands
>
> T: +31(0)50-3615002
> F: +31(0)50-3614862
> E: m.vugt@umcg.nl

From: **Sergi Guerrero** sergibiotec@gmail.com
Subject: Re: Authorship request....
Date: 9 September 2020 at 12:03
To: Vugt, MATM van m.vugt@umcg.nl
Cc: Kok, YP (onco) y.p.kok@umcg.nl, Schoonen, PM p.m.schoonen@umcg.nl, Everts, M evertsm@umcg.nl, Bhattacharya, A (onco) a.bhattacharya@umcg.nl, Fehrmann, RSN (int) r.s.n.fehrmann@umcg.nl, Tempel, N van den (onco) n.van.den.tempel@umcg.nl

---

I agree

On Wed, Sep 9, 2020, 12:02 Vugt, MATM van <m.vugt@umcg.nl> wrote:

> Dear all,
>
> I just sent you an email with a request to agree with the new authorship. This is the exact same authorship change that I asked you to agree on before, but the old/new authorship needed to be included.
>
> Could you please reply to that email by saying that you agree? Thanks for your help!
>
> Marcel
>
> Could you please reply to this email if you agree with the updated authorship for the manuscript 'Overexpression of Cyclin E1 or Cdc25A leads to replication stress, mitotic aberrancies and increased sensitivity to replication checkpoint inhibitors'.
>
> Original authorship (with Sergi Guerrero Llobet and Pepijn Schoonen sharing first authorship):
>
> Sergi Guerrero Llobet[1, *], Pepijn M. Schoonen[1, *], Marieke Everts[1], Yannick P. Kok[1], Nathalie van den Tempel[1], Marcel A.T.M. van Vugt[1,2]
>
> New authorship (with Yannick Kok, Sergi Guerrero Llobet and Pepijn Schoonen sharing first authorship, and Nathalie van den Tempel and Marcel Van Vugt sharpen senior authorship and correspondence)?
>
> Yannick P. Kok[1,*], Sergi Guerrero Llobet[1,*], Pepijn M. Schoonen[1,*], Marieke Everts[1], Arkajyoti Bhattacharya[1], Rudolf S.N. Fehrmann[1], Nathalie van den Tempel[1,*,], Marcel A.T.M. van Vugt[1,*]
>
> Kind regards, Marcel
>
> --
> Marcel A.T.M. van Vugt, PhD
>
> Department of Medical Oncology
> University Medical Center Groningen
> PO box 30.001
> Room F1.23/DA13
> Hanzeplein 1
> 9700 RB Groningen
> The Netherlands
>
> T: +31(0)50-3615002
> F: +31(0)50-3614862
> E: m.vugt@umcg.nl
>
> ---
>
> De inhoud van dit bericht is vertrouwelijk en alleen bestemd voor de geadresseerde(n). Anderen dan de geadresseerde(n) mogen geen gebruik maken van dit bericht, het niet openbaar maken of op enige wijze verspreiden of vermenigvuldigen. Het UMCG kan niet aansprakelijk gesteld worden voor een incomplete aankomst of vertraging van dit verzonden bericht.
>
> The contents of this message are confidential and only intended for the eyes of the addressee(s). Others than the addressee(s) are not allowed to use this message, to make it public or to distribute or multiply this message in any way. The UMCG cannot [illegible]

be held responsible for incomplete reception or delay of this transferred message.

From: **Schoonen, PM** p.m.schoonen@umcg.nl
Subject: Re: Authorship request....
Date: 9 September 2020 at 12:37
To: Bhattacharya, A (onco) a.bhattacharya@umcg.nl, Sergi Guerrero sergibiotec@gmail.com
Cc: Vugt, MATM van m.vugt@umcg.nl, Kok, YP (onco) y.p.kok@umcg.nl, Everts, M evertsm@umcg.nl, Fehrmann, RSN (int) r.s.n.fehrmann@umcg.nl, Tempel, N van den (onco) n.van.den.tempel@umcg.nl

---

I agree.

Best,
Pepijn

Van: Bhattacharya, A (onco)
Verzonden: woensdag 9 september 2020 12:31:01
Aan: Sergi Guerrero
CC: Vugt, MATM van; Kok, YP (onco); Schoonen, PM; Everts, M; Fehrmann, RSN (int); Tempel, N van den (onco)
Onderwerp: Re: Authorship request....

I agree.

Regards,
Arkajyoti

> On 9 Sep 2020, at 12:02, Sergi Guerrero <sergibiotec@gmail.com> wrote:
>
> I agree
>
> On Wed, Sep 9, 2020, 12:02 Vugt, MATM van <m.vugt@umcg.nl> wrote:
>
>> Dear all,
>>
>> I just sent you an email with a request to agree with the new authorship. This is the exact same authorship change that I asked you to agree on before, but the old/new authorship needed to be included.
>>
>> Could you please reply to that email by saying that you agree? Thanks for your help!
>>
>> Marcel
>>
>> Could you please reply to this email if you agree with the updated authorship for the manuscript 'Overexpression of Cyclin E1 or Cdc25A leads to replication stress, mitotic aberrancies and increased sensitivity to replication checkpoint inhibitors'.
>>
>> Original authorship (with Sergi Guerrero Llobet and Pepijn Schoonen sharing first authorship):
>>
>> Sergi Guerrero Llobet[1, *], Pepijn M. Schoonen[1, *], Marieke Everts[1], Yannick P. Kok[1], Nathalie van den Tempel[1], Marcel A.T.M. van Vugt[1,2]
>>
>> New authorship (with Yannick Kok, Sergi Guerrero Llobet and Pepijn Schoonen sharing first authorship, and Nathalie van den Tempel and Marcel Van Vugt sharpen senior authorship and correspondence)?
>>
>> Yannick P. Kok[1,*], Sergi Guerrero Llobet[1,*], Pepijn M. Schoonen[1,*], Marieke Everts[1], Arkajyoti Bhattacharya[1], Rudolf S.N. Fehrmann[1], Nathalie van den Tempel[1,*,], Marcel A.T.M. van Vugt[1,*]
>>
>> Kind regards, Marcel
>>
>> --
>> Marcel A.T.M. van Vugt, PhD
>>
>> Department of Medical Oncology
>> University Medical Center Groningen
>> PO box 30.001
>> Room F1.23/DA13
>> [illegible]

Hanzeplein 1
9700 RB Groningen
The Netherlands

T: +31(0)50-3615002
F: +31(0)50-3614862
E: m.vugt@umcg.nl

De inhoud van dit bericht is vertrouwelijk en alleen bestemd voor de geadresseerde(n). Anderen dan de geadresseerde(n) mogen geen gebruik maken van dit bericht, het niet openbaar maken of op enige wijze verspreiden of vermenigvuldigen. Het UMCG kan niet aansprakelijk gesteld worden voor een incomplete aankomst of vertraging van dit verzonden bericht.

The contents of this message are confidential and only intended for the eyes of the addressee(s). Others than the addressee(s) are not allowed to use this message, to make it public or to distribute or multiply this message in any way. The UMCG cannot be held responsible for incomplete reception or delay of this transferred message.

From: **Tempel, N van den (onco)** n.van.den.tempel@umcg.nl
Subject: Re: Oncogenesis authorship
Date: 9 September 2020 at 12:00
To: Vugt, MATM van m.vugt@umcg.nl

Dear Marcel,

I agree with the updated authorship for this manuscript.

Kind regards,
Nathalie van den Tempel

From: Vugt, MATM van
Sent: Wednesday, September 9, 2020 11:59:28 AM
To: Kok, YP (onco); Sergi Guerrero; Schoonen, PM; Everts, M; Bhattacharya, A (onco); Fehrmann, RSN (int); Tempel, N van den (onco)
Subject: Oncogenesis authorship

Dear co-authors,

Could you please reply to this email if you agree with the updated authorship for the manuscript 'Overexpression of Cyclin E1 or Cdc25A leads to replication stress, mitotic aberrancies and increased sensitivity to replication checkpoint inhibitors'.

Original authorship (with Sergi Guerrero Llobet and Pepijn Schoonen sharing first authorship):
Sergi Guerrero Llobet[1, *], Pepijn M. Schoonen[1, *], Marieke Everts[1], Yannick P. Kok[1], Nathalie van den Tempel[1], Marcel A.T.M. van Vugt[1,2]

New authorship (with Yannick Kok, Sergi Guerrero Llobet and Pepijn Schoonen sharing first authorship, and Nathalie van den Tempel and Marcel Van Vugt sharpen senior authorship and correspondence)?
Yannick P. Kok[1,*], Sergi Guerrero Llobet[1,*], Pepijn M. Schoonen[1,*], Marieke Everts[1], Arkajyoti Bhattacharya[1], Rudolf S.N. Fehrmann[1], Nathalie van den Tempel[1,*,], Marcel A.T.M. van Vugt[1,*]

Kind regards, Marcel

Marcel A.T.M. van Vugt, PhD

Department of Medical Oncology
University Medical Center Groningen
PO box 30.001
Room F1.23/DA13
Hanzeplein 1
9700 RB Groningen
The Netherlands

T: +31(0)50-3615002
F: +31(0)50-3614862
E: m.vugt@umcg.nl

From: **Bhattacharya, A (onco)** a.bhattacharya@umcg.nl
Subject: Re: Authorship request....
Date: 9 September 2020 at 12:31
To: Sergi Guerrero sergibiotec@gmail.com
Cc: Vugt, MATM van m.vugt@umcg.nl, Kok, YP (onco) y.p.kok@umcg.nl, Schoonen, PM p.m.schoonen@umcg.nl, Everts, M evertsm@umcg.nl, Fehrmann, RSN (int) r.s.n.fehrmann@umcg.nl, Tempel, N van den (onco) n.van.den.tempel@umcg.nl

---

I agree.

Regards,
Arkajyoti

> On 9 Sep 2020, at 12:02, Sergi Guerrero <sergibiotec@gmail.com> wrote:
>
> I agree
>
> On Wed, Sep 9, 2020, 12:02 Vugt, MATM van <m.vugt@umcg.nl> wrote:
>
>> Dear all,
>>
>> I just sent you an email with a request to agree with the new authorship. This is the exact same authorship change that I asked you to agree on before, but the old/new authorship needed to be included.
>>
>> Could you please reply to that email by saying that you agree? Thanks for your help!
>>
>> Marcel
>>
>> Could you please reply to this email if you agree with the updated authorship for the manuscript 'Overexpression of Cyclin E1 or Cdc25A leads to replication stress, mitotic aberrancies and increased sensitivity to replication checkpoint inhibitors'.
>>
>> Original authorship (with Sergi Guerrero Llobet and Pepijn Schoonen sharing first authorship):
>>
>> Sergi Guerrero Llobet[1, *], Pepijn M. Schoonen[1, *], Marieke Everts[1], Yannick P. Kok[1], Nathalie van den Tempel[1], Marcel A.T.M. van Vugt[1,2]
>>
>> New authorship (with Yannick Kok, Sergi Guerrero Llobet and Pepijn Schoonen sharing first authorship, and Nathalie van den Tempel and Marcel Van Vugt sharpen senior authorship and correspondence)?
>>
>> Yannick P. Kok[1,*], Sergi Guerrero Llobet[1,*], Pepijn M. Schoonen[1,*], Marieke Everts[1], Arkajyoti Bhattacharya[1], Rudolf S.N. Fehrmann[1], Nathalie van den Tempel[1,*], Marcel A.T.M. van Vugt[1,*]
>>
>> Kind regards, Marcel
>>
>> ---
>>
>> Marcel A.T.M. van Vugt, PhD
>>
>> Department of Medical Oncology
>> University Medical Center Groningen
>> PO box 30.001
>> Room F1.23/DA13
>> Hanzeplein 1
>> 9700 RB Groningen
>> The Netherlands
>>
>> T: +31(0)50-3615002
>> F: +31(0)50-3614862
>> E: m.vugt@umcg.nl
>>
>> ---
>>
>> [illegible]

De inhoud van dit bericht is vertrouwelijk en alleen bestemd voor de geadresseerde(n). Anderen dan de geadresseerde(n) mogen geen gebruik maken van dit bericht, het niet openbaar maken of op enige wijze verspreiden of vermenigvuldigen. Het UMCG kan niet aansprakelijk gesteld worden voor een incomplete aankomst of vertraging van dit verzonden bericht.

The contents of this message are confidential and only intended for the eyes of the addressee(s). Others than the addressee(s) are not allowed to use this message, to make it public or to distribute or multiply this message in any way. The UMCG cannot be held responsible for incomplete reception or delay of this transferred message.

From: **Everts, M** evertsm@umcg.nl
Subject: Re: Oncogenesis authorship
Date: 9 September 2020 at 12:34
To: Vugt, MATM van m.vugt@umcg.nl, Kok, YP (onco) y.p.kok@umcg.nl, Sergi Guerrero sergibiotec@gmail.com, Schoonen, PM p.m.schoonen@umcg.nl, Bhattacharya, A (onco) a.bhattacharya@umcg.nl, Fehrmann, RSN (int) r.s.n.fehrmann@umcg.nl, Tempel, N van den (onco) n.van.den.tempel@umcg.nl

I agree.

Gr, Marieke

Outlook voor iOS downloaden

Van: Vugt, MATM van <m.vugt@umcg.nl>
Verzonden: Wednesday, September 9, 2020 11:59:28 AM
Aan: Kok, YP (onco) <y.p.kok@umcg.nl>; Sergi Guerrero <sergibiotec@gmail.com>; Schoonen, PM <p.m.schoonen@umcg.nl>; Everts, M <evertsm@umcg.nl>; Bhattacharya, A (onco) <a.bhattacharya@umcg.nl>; Fehrmann, RSN (int) <r.s.n.fehrmann@umcg.nl>; Tempel, N van den (onco) <n.van.den.tempel@umcg.nl>
Onderwerp: Oncogenesis authorship

Dear co-authors,

Could you please reply to this email if you agree with the updated authorship for the manuscript 'Overexpression of Cyclin E1 or Cdc25A leads to replication stress, mitotic aberrancies and increased sensitivity to replication checkpoint inhibitors'.

Original authorship (with Sergi Guerrero Llobet and Pepijn Schoonen sharing first authorship):

Sergi Guerrero Llobet[1, *], Pepijn M. Schoonen[1, *], Marieke Everts[1], Yannick P. Kok[1], Nathalie van den Tempel[1], Marcel A.T.M. van Vugt[1,2]

New authorship (with Yannick Kok, Sergi Guerrero Llobet and Pepijn Schoonen sharing first authorship, and Nathalie van den Tempel and Marcel Van Vugt sharpen senior authorship and correspondence)?

Yannick P. Kok[1,*], Sergi Guerrero Llobet[1,*], Pepijn M. Schoonen[1,*], Marieke Everts[1], Arkajyoti Bhattacharya[1], Rudolf S.N. Fehrmann[1], Nathalie van den Tempel[1,*,], Marcel A.T.M. van Vugt[1,*]

Kind regards, Marcel

Marcel A.T.M. van Vugt, PhD

Department of Medical Oncology
University Medical Center Groningen
PO box 30.001
Room F1.23/DA13
Hanzeplein 1
9700 RB Groningen
The Netherlands

T: +31(0)50-3615002
F: +31(0)50-3614862
E: m.vugt@umcg.nl
